# Structural and functional analysis of LarC, a CTP-dependent cyclometallase required for nickel-pincer nucleotide cofactor biosynthesis

**DOI:** 10.1126/sciadv.aeh6867

**Published:** 2026-08-26

**Authors:** Robert Wolfe, Aiko Turmo, Samantha Velasquez-Rivertte, Dexin Sui, Diego Granados-Villanueva, Benoit Desguin, Jian Hu, Robert P. Hausinger, Kelly H. Kim

**Affiliations:** ^1^ Department of Biochemistry and Molecular Biology, Michigan State University, East Lansing, Michigan, USA.; ^2^ Department of Microbiology, Genetics, and Immunology, Michigan State University, East Lansing, Michigan, USA.; ^3^ Louvain Institute of Biomolecular Science and Technology (LIBST), Université catholique de Louvain, Louvain-La-Neuve, Belgium.; ^4^ Department of Chemistry, Michigan State University, East Lansing, Michigan, USA.

## Abstract

Biosynthesis of the nickel-pincer mononucleotide metallocofactor requires a CTP-dependent nickel insertion reaction catalyzed by LarC, whose mechanism of C-Ni bond formation is not fully understood. Here, we report the first cryo–electron microscopy structures of full-length LarC from *Moorella thermoacetica* with and without a mimic of the CMPylated reaction intermediate. LarC assembles as a hexamer comprising a central LarC2 domain core and peripheral LarC1 domain trimers connected by long, flexible interdomain linkers. The LarC1 domains contain a conserved histidine-rich region for nickel binding and an adjacent conserved acidic pocket, both essential for activity. Structural modeling suggests that the intermediate binds within the acidic pocket adjacent to the putative nickel-binding site, while cryo-EM density for an intermediate analog identifies an interdomain cleft near the LarC2 CTP-binding site as a likely transfer site. Based on these findings, we propose that the intermediate is transferred through the interdomain cleft from LarC2, where it is CMPylated, to LarC1 for nickel insertion.

## INTRODUCTION

**Fig. 1.**
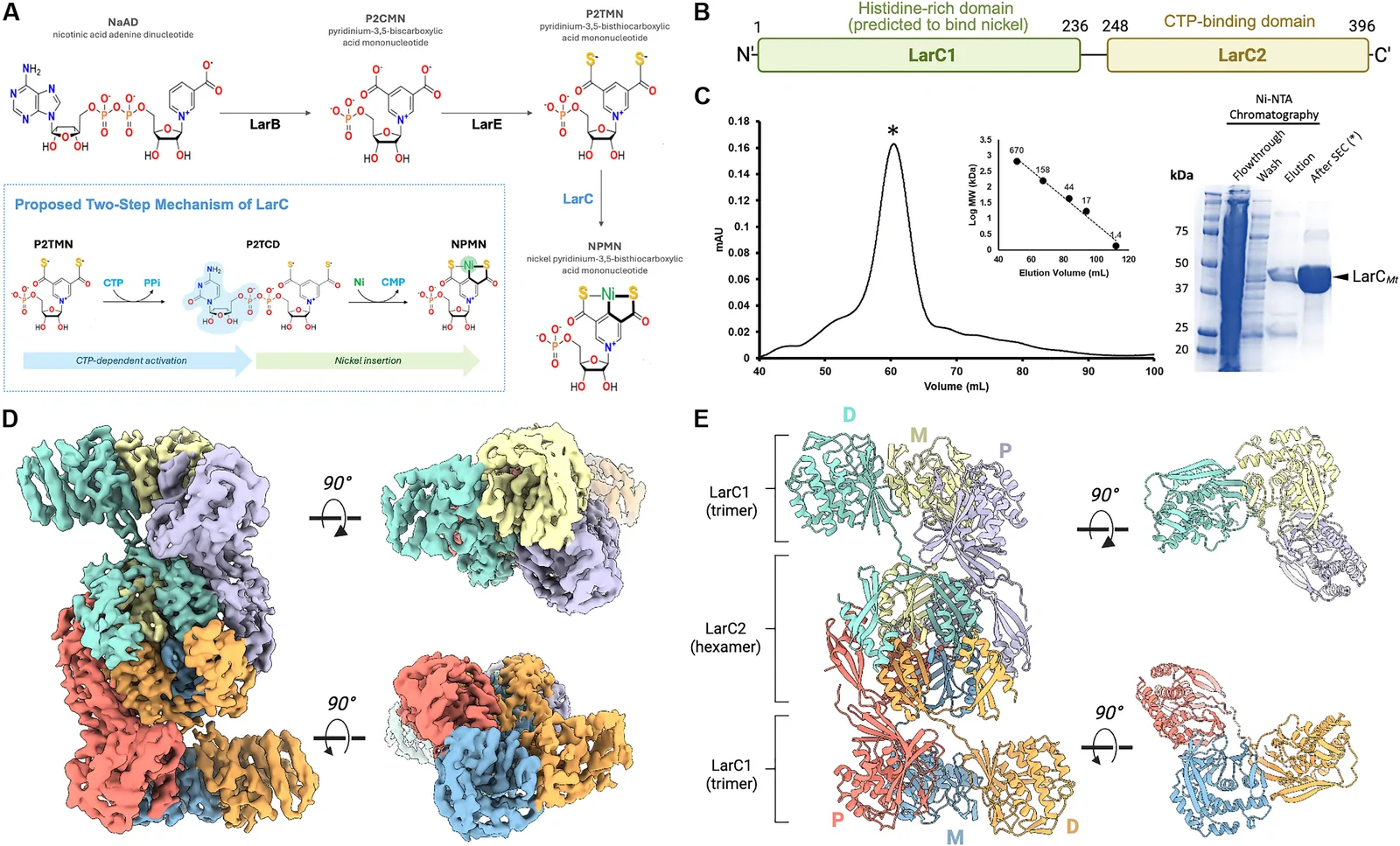
Overall architecture and domain organization of full-length LarC. (**A**) Schematic of nickel pincer mononucleotide (NPMN) cofactor biosynthesis. NaAD is converted to P2CMN by LarB through ring carboxylation and hydrolysis. P2CMN is converted to P2TMN by LarE via double sulfur insertion. LarC catalyzes the final stage of cofactor maturation by inserting nickel into P2TMN to generate the mature nickel pincer mononucleotide cofactor NPMN. The inset illustrates the proposed two step LarC mechanism, in which P2TMN is first CMPylated to form the P2TCD intermediate, followed by nickel insertion and CMP release to yield NPMN. (**B**) Domain organization of LarC*_Mt_*. (**C**) Size-exclusion chromatography (SEC) profile of purified LarC*_Mt,_* [adapted from fig. S3C in Turmo *et al.* (*8*)] with corresponding SDS-PAGE analysis. The inset depicts the elution positions for standards of the indicated sizes. (**D**) Cryo-EM reconstruction of full-length LarC*_Mt_* revealing a hexameric assembly, with each monomer shown in a different color. (**E**) Atomic model of the LarC*_Mt_* hexamer fit into the cryo-EM density, highlighting the central LarC2 hexameric core and flanking LarC1 trimers. The three LarC1 protomers within each trimer are labeled as proximal (P), middle (M), and distal (D) based on their relative proximity to the central LarC2 hexamer. Colored the same as in D.

Nickel is an essential trace element required by many bacteria and archaea for the function of various enzymes, which are crucial in metabolic processes such as energy production and detoxification (*1*). Among these, the nickel-pincer mononucleotide (NPMN), discovered in lactate racemase (*2*, *3*), has emerged as a critical cofactor in various racemase, epimerase, and other reactions (*4*). In lactate racemase, the structure of the NPMN cofactor has a nickel ion coordinated to pyridinium-3,5-bisthiocarboxylic acid mononucleotide (P2TMN) (*3*) (Fig. 1A).

The biosynthesis of the NPMN cofactor is a highly specialized process involving a series of enzymatic steps carried out by LarB, LarE, and LarC (*5*, *6*) (Fig. 1A). While LarB and LarE are responsible for production of P2TMN (*7*), LarC executes the final step of inserting Ni^2+^ into the organic framework, thereby completing the synthesis of the mature NPMN cofactor (*8*, *9*).

LarC is distinguished as the first and only known cyclometallase in nature, catalyzing the formation of a Ni-C covalent bond within the NPMN structure (*9*) (Fig. 1A). This reaction is exceptionally challenging under physiological conditions due to the high p*K*_a_ of the carbon atom involved (*6*, *10*). This novel reaction not only underscores the enzyme’s significance in the broader context of biochemistry but also expands our understanding of how nature manipulates metal ions to create biologically active cofactors (*11*). The discovery of LarC has opened new avenues for exploring metal insertion mechanisms, making it a subject of great interest in biochemical research (*8*, *12*).

The precise molecular mechanism of LarC is not yet fully understood; however, previous studies, primarily using the *Lactiplantibacillus plantarum* enzyme (LarC*_Lp_*), have revealed several of its intriguing properties (*8*, *9*). Structurally, LarC*_Lp_* is organized into two distinct domains, with LarC1 (residues 1–271) and LarC2 (residues 272–412) domains encoded by distinct open reading frames separated by a stop codon that is bypassed using a programmed ribosomal frameshift (*9*, *13*) (Fig. 1B). Within LarC1 lies a region containing several conserved histidine residues that are thought to be responsible for nickel binding (*9*, *12*) (fig. S1). On the other hand, LarC2 contains the binding site for CTP, which is essential for LarC’s catalytic function (*5*, *9*, *12*). The crystal structure of the latter domain of LarC*_Lp_* revealed a hexamer with six CTP molecules bound in deep clefts formed at the subunit interfaces (*9*). Substituting residues involved in CTP-binding reduces LarC’s nickel insertase activity, but the reason for CTP’s necessity remained unclear until a mass spectrometry study was performed using the enzyme from *Moorella thermoacetica* (LarC*_Mt_*) (*8*). The researchers found that a CMPylated PT2MN intermediate, pyridinium-bisthiocarboxylic acid cytidine dinucleotide (P2TCD), is generated during the enzymatic reaction that was dependent on CTP and increased in level when nickel ions were limiting (*8*). These observations suggest that CTP is required for the intermediate’s formation and that nickel is essential for its conversion into the final product, the NPMN cofactor (*5*, *8*, *9*, *12*). The nickel insertion step is accompanied by hydrolysis of the phosphoanhydride to release CMP (Fig. 1A). Overall, these findings established a foundational understanding of LarC’s enzymatic function and structural organization.

The P2TCD intermediate is reminiscent of the intermediate formed by an ATP-dependent molybdenum insertase, Cnx1, that synthesizes the molybdenum cofactor (Moco) (*14*–*16*). In that case, the substrate molybdopterin is thought to be adenylylated to properly position it near the molybdenum-binding site of Cnx1 (*14*, *16*). This intermediate then undergoes metal insertion, followed by cleavage with release of AMP and the final product, Moco (*16*). We hypothesize that a similar process occurs during NPMN cofactor biosynthesis, where P2TMN is CMPylated to properly orient it near the nickel-binding site, followed by nickel insertion and cleavage to release CMP and the NPMN cofactor.

Despite advances in understanding LarC’s function and mechanism, several critical knowledge gaps persist. A major deficiency is the lack of a full-length structure of the enzyme, thus limiting insights into domain interactions during catalysis. Additionally, the binding site for the P2TMN and nickel substrates and the precise positioning of the P2TCD intermediate within LarC remain unclear. There is also limited understanding of the dynamic conformational changes that LarC undergoes during the catalytic cycle, particularly how these movements coordinate the binding of CTP, nickel, and P2TMN, and the subsequent steps leading to product formation. Furthermore, while mutagenesis studies have provided insights into a few key residues involved in activity (*9*), a detailed mechanistic understanding of their roles in the reaction pathway remains elusive. These challenges underscore the need for further structural and biochemical studies to fully elucidate LarC’s complex catalytic mechanism.

To address these knowledge gaps, we determined the first cryo–electron microscopy (cryo-EM) structure of a LarC using the intact LarC*_Mt_* enzyme, both in the presence and absence of an intermediate analog, pyridinium-3,5-bisthiocarboxylic acid adenine dinucleotide (P2TAD), which mimics the P2TCD intermediate except it is AMPylated rather than CMPylated (*17*). This structural knowledge provides a comprehensive view of the full-length LarC, revealing the spatial arrangement, conformational dynamics, and interactions between the LarC1 and LarC2 domains. Our cryo-EM structures, accompanied by computational protein-ligand structural prediction, also allowed us to identify a potential P2TCD-binding site, giving us new insights into how LarC interacts with its substrates during catalysis. These results provide a foundation for further exploration into the enzyme’s unique mechanism of nickel insertion and its role in NPMN biosynthesis.

## RESULTS

### Cryo-EM structure of full-length LarC from *M. thermoacetica* reveals a unique hexameric assembly

We screened several LarC homologs to obtain an intact, full-length protein because the purified enzyme from *L. plantarum* underwent proteolytic degradation to produce separate LarC1 and LarC2 components. The LarC*_Mt_* version produced the highest yield and optimal homogeneity and was therefore selected for subsequent structural studies.

The gene encoding LarC*_Mt_* was cloned with the sequence for an N-terminal His_6_-tag, overexpressed in *Escherichia coli* BL21(DE3), and purified using nickel-nitrilotriacetic acid (Ni-NTA) affinity chromatography followed by size-exclusion chromatography (SEC). The SEC chromatogram showed that the protein eluted earlier than expected for a 42 kDa protein (Fig. 1C), suggesting formation of a higher-order oligomer, consistent with the hexameric assembly observed previously for the isolated LarC2 domain from *L. plantarum* (*9*).

Purified LarC*_Mt_* was homogeneous and monodisperse, as judged by the SEC profiles, and was subjected to structure determination by cryo-EM. The cryo-EM reconstruction of full-length LarC*_Mt_* was determined at 3.97 Å resolution and confirmed a hexameric assembly (Fig. 1, D and E and figs. S2 and S3). The central core of the hexamer is formed by the LarC2 domains, which adopt a fold consistent with previous crystal structures (*9*) (Fig. 1, D and E and fig. S2). Each LarC2 monomer contains an N-terminal region with a ferredoxin-like fold and a C-terminal region that extends outward to form a protrusion contributing to the deep CTP-binding pocket. In the hexamer, two trimers of the LarC2 N-terminal region stack along a three-fold symmetry axis to form a dimer-of-trimers assembly, while the LarC2 C-terminal regions radiate from the core (Fig. 1E). The LarC2 architecture observed in our cryo-EM structure closely resembles that seen in previously published crystal structures (*9*) (fig. S2, A and B), confirming that the hexameric arrangement and domain organization are preserved in the full-length protein that shares 38% sequence identity with LarC*_Lp_*.

Extending from the N terminus of each LarC2 monomer is a 13-residue flexible linker that connects to the N-terminal LarC1 domain, which had not been visualized previously (Fig. 1, D and E). Each LarC1 monomer adopts a mixed α/β architecture, consisting of a seven-helix bundle juxtaposed against a seven-stranded antiparallel β-sheet (Fig. 1E). Structural similarity searches using DALI (*18*) and Foldseek (*19*) failed to identify any close structural homologs of the LarC1 domain among proteins of known structure, with only weak matches to diverse and functionally unrelated proteins, indicating that LarC1 adopts a previously uncharacterized protein fold.

Three LarC1 monomers assemble in a head-to-tail arrangement to form a V-shaped trimer, with one trimer flanking each side of the central LarC2 hexamer (Fig. 1, D and E). Unlike the symmetric LarC2 hexamer, the LarC1 trimer adopts an asymmetric architecture in which the three LarC1 protomers occupy distinct positions relative to the LarC2 core. Accordingly, the protomers can be distinguished as proximal, middle, and distal based on their relative proximity to the LarC2 hexamer core (Fig. 1E and fig. S2, E and F). Within each LarC1 trimer, two distinct pairwise interaction interfaces are formed. Each interface buries an average of 895 Å^2^ and is stabilized by van der Waals and hydrogen bond interactions (fig. S2, C and D). While interface residues are not uniformly conserved, multiple positions, including V107, H108, R231, and P166, display strong conservation across LarC homologs (figs. S1 and S2D).

Together, the LarC1 trimeric assemblies and the LarC2 hexameric core define the architecture of the full-length LarC hexamer, with the LarC2 core sandwiched between the two LarC1 trimers.

### Putative nickel-binding site in the LarC1 domain

**Fig. 2.**
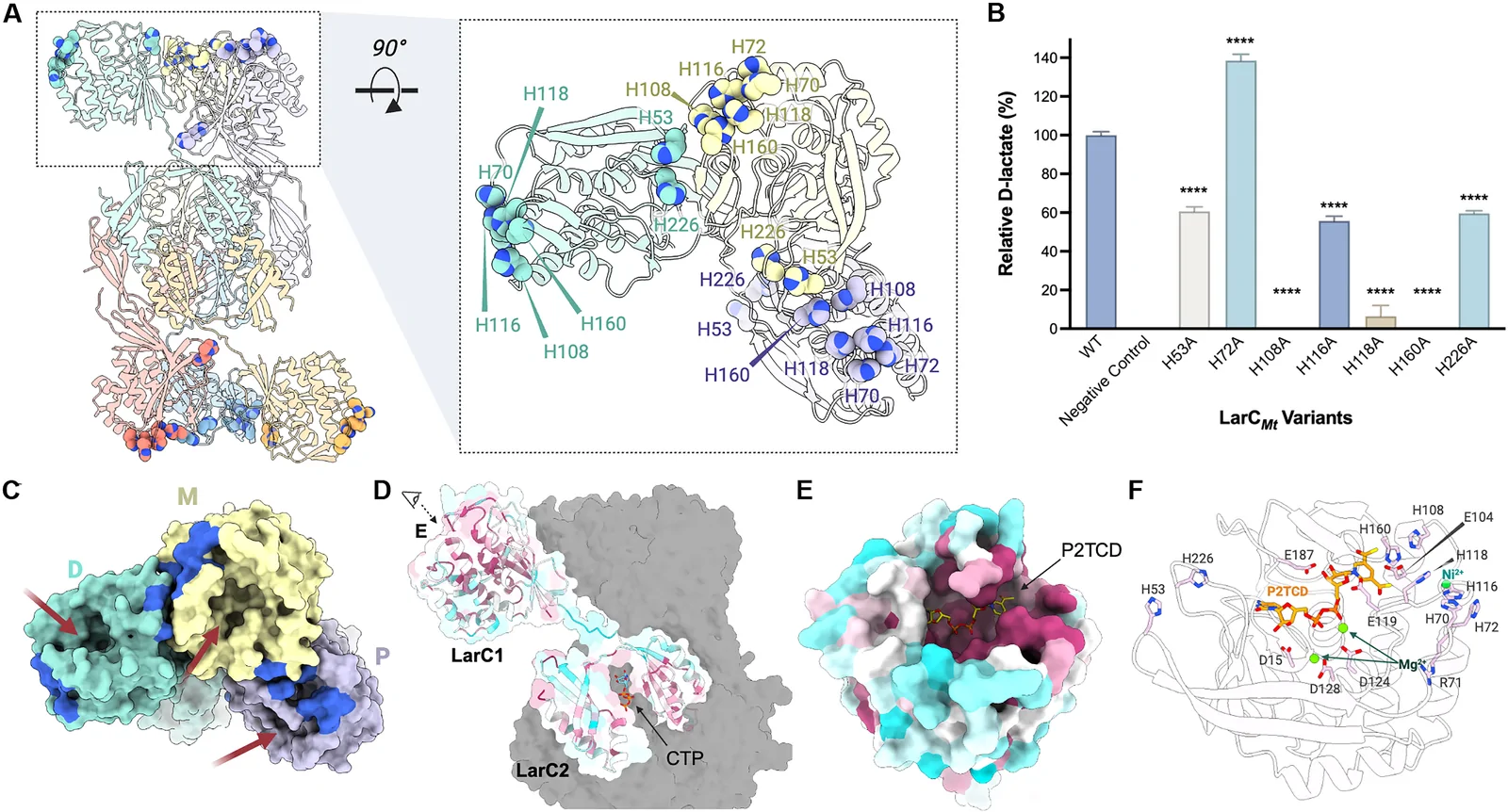
Putative nickel and P2TCD binding sites in the LarC1 domain. (**A**) Structure of the LarC1 domain highlighting a surface-exposed histidine-rich region formed at the LarC1-LarC1 interface. Histidine residues are shown as spheres on the ribbon representation. (**B**) Effects of alanine substitutions within the histidine-rich region on LarC activity, as measured by lactate racemization assay. Statistical significance was assessed using one-way ANOVA followed by Dunnett’s multiple comparisons test (****P < 0.0001). Each data point represents a technical replicate (*n* = 3 assay replicates per variant). (**C**) Surface representation of the LarC1 domain showing a deep pocket adjacent to the histidine-rich surface. Histidine residues are shown in blue, and arrows point to the pocket in each monomer. The three LarC1 protomers within each trimer are labeled as proximal (P), middle (M), and distal (D) based on their relative proximity to the central LarC2 hexamer. (**D**) Sequence conservation mapped onto the LarC*_Mt_* structure, with maroon indicating highly conserved residues and blue indicating variable residues. For clarity, a single LarC monomer is shown in color, while the remaining subunits are rendered in grey. The CTP molecule is shown for reference at the CTP binding site (not present in this structure; modeled from the CTP-bound LarC2*_Lp_* crystal structure, PDB: 6BWQ). (**E**) Sequence conservation mapped onto the LarC1 domain, highlighting that the pocket region corresponds to one of the most highly conserved surface features. The predicted position of P2TCD is shown. (**F**) Predicted model of P2TCD bound within the conserved LarC1 pocket, illustrating a potential substrate binding mode.

Previous studies have suggested that conserved histidine residues in LarC play a key role in nickel binding (*5*, *9*). Although the LarC1 domain of LarC*_Mt_* contains fewer histidine residues than that of LarC*_Lp_* (which contains 21 histidines), it still harbors eight (H53, H70, H72, H108, H116, H118, H160, H226), with five (H72, H108, H116, H118, and H160) conserved in position between LarC*_Lp_* and LarC*_Mt_* (fig. S1). Interestingly, despite being separated in the sequence, these residues are found clustered together in our cryo-EM structure. Together, these residues form a surface-exposed patch on each LarC1 monomer that faces away from the LarC2 hexamer and lies adjacent to the neighboring LarC1 monomer (Fig. 2A). H70, H72, H108, H116, H118, and H160 from one monomer align next to H53 and H226 of the neighboring monomer, generating a continuous, solvent-exposed histidine-rich surface across the LarC1-LarC1 interface (Fig. 2A).

Given the close spatial arrangement of these residues and prior evidence implicating them in nickel binding, we examined this region for electron density corresponding to a bound nickel ion. Although NiCl_2_ was supplied during cell expression and this metal ion was added to the purified sample prior to cryo-EM grid preparation, no discernible nickel density was observed in the cryo-EM map. This absence may reflect low nickel occupancy under our experimental conditions and/or limited local resolution in this relatively flexible region compared with the more rigid core of the complex (fig. S3).

While nickel was not detected in our structure, we evaluated the functional relevance of this histidine cluster by generating single alanine substitutions and assessing their impact on NPMN cofactor biosynthesis using the lactate racemization assay. In this assay, LarA*_Lp_* with a Strep-tag II was co-expressed with LarB*_Lp_*, LarE*_Lp_*, and either wild-type or variant LarC*_Mt_*. All substitutions except H72A led to substantial loss of LarA*_Lp_* activity. Mutations leading to changes at H53, H116, and H226 were moderately debilitating (∼60% of wild-type activity), whereas the H108A, H118A, and H160A substitutions nearly abolished activity (<10% of that for the wild-type enzyme) (Fig. 2B). Collectively, these findings reinforce the notion that this surface-exposed histidine cluster is indispensable for LarC activity and most likely forms the nickel-binding site(s) required for NPMN cofactor biosynthesis, as suggested by earlier work (*8*, *9*).

### Putative CMPylated-substrate-binding site

Our cryo-EM structure shows that each LarC1 domain contains a deep pocket on its outer face, adjacent to the histidine-rich patch (Fig. 2C). Each LarC1 trimer therefore contains three pockets, and since LarC2 hexamer is flanked by two LarC1 trimers on opposite sides, the LarC hexamer has a total of six pockets. In both trimers, all three pockets are oriented away from the central LarC2 domain. Nevertheless, the asymmetric organization of the LarC1 trimer positions the pockets of the proximal, middle, and distal LarC1 protomers at distinct distances and orientations relative to the LarC2 hexamer (Fig. 2C and fig. S2E). Despite these positional differences, inspection of the pocket surfaces revealed a common environment in which all six pockets are lined with conserved acidic residues (Fig. 2, D and E), some previously shown to be important for LarC*_Lp_* catalysis.

To assess whether this pocket could plausibly accommodate the LarC substrate for nickel insertion (i.e., P2TCD), we used the Chai-1 server (*20*) to generate a model of LarC with this bound reaction intermediate (Fig. 1A). In this model, P2TCD is positioned within the conserved pocket, with its pyridinium ring oriented toward the suspected nickel-binding site and E104 (Fig. 2, E and F), which is equivalent to E124 in *L. plantarum* LarC and whose substitution has been shown to abolish activity (*9*), suggesting a possibility that this region may function as a catalytic site for nickel insertion. However, because these pockets are oriented away from the CTP binding site in the LarC2 domain (Fig. 2D), it remains unclear how P2TMN, which presumably undergoes CMPylation in LarC2, is subsequently transferred across the enzyme to reach the conserved LarC1 pocket for the nickel insertion step.

**Fig. 3.**
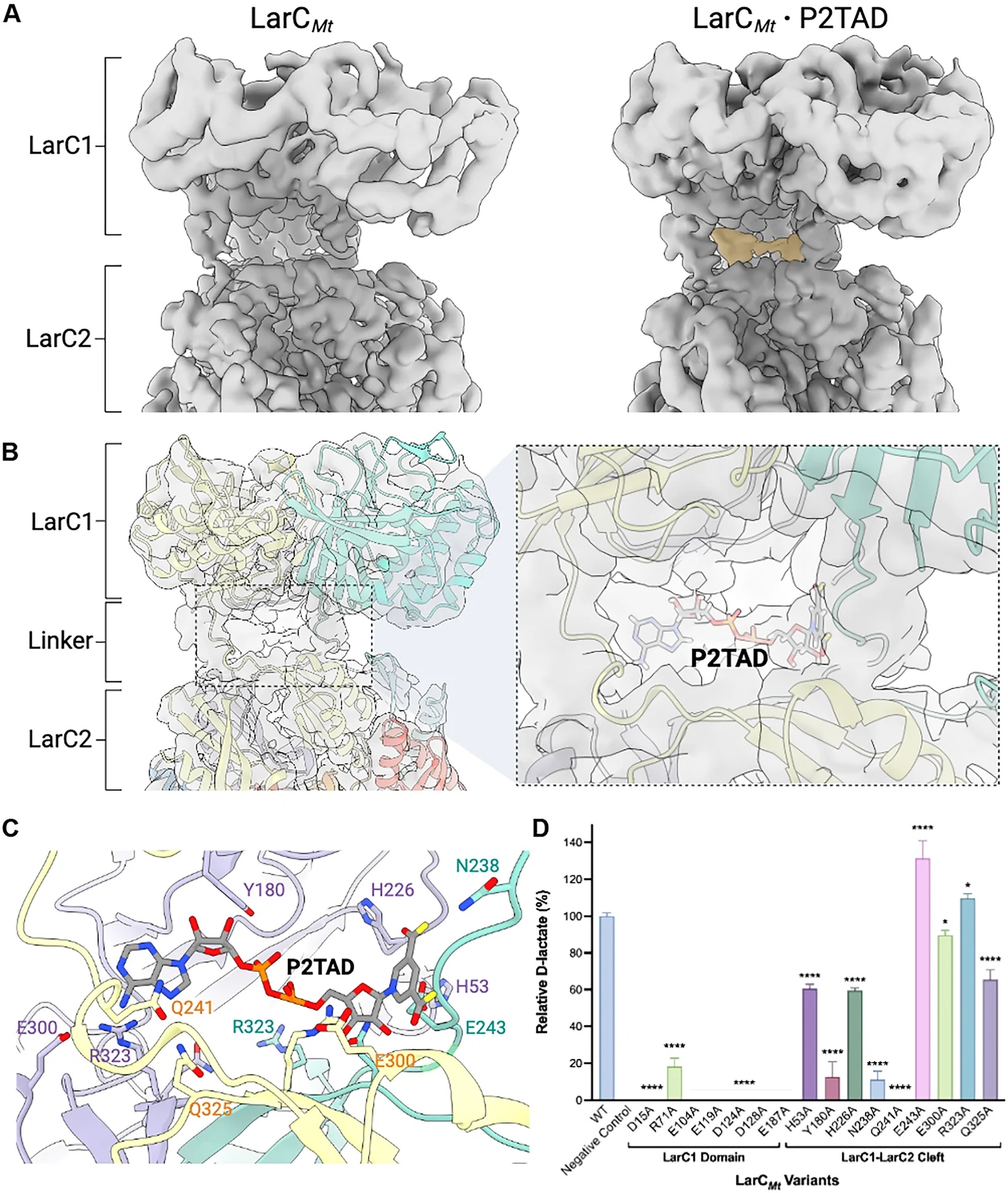
Putative binding sites for the intermediate of LarC. (**A**) Comparison of cryo-EM density maps of LarC*_Mt_* in the absence (left) and presence (right) of P2TAD shows additional density (orange) in the LarC1-LarC2 interdomain cleft in the LarC*_Mt_*●P2TAD complex. (**B**) Cryo-EM map with the atomic model fitted. The inset shows a close-up view of the extra density in the LarC1–LarC2 cleft with P2TAD fitted into this region. (**C**) Close-up view of the interdomain cleft highlighting residues surrounding the extra density corresponding to the putative P2TAD binding site. (**D**) Effects of alanine substitutions in residues lining the conserved LarC1 pocket and the LarC1-LarC2 cleft on LarC activity, measured by lactate racemization assays. Statistical significance was assessed using one-way ANOVA followed by Dunnett’s multiple comparisons test (****P < 0.0001; *P < 0.05). Each data point represents a technical replicate (*n* = 3 assay replicates per variant).

To further explore P2TCD binding, we determined a cryo-EM structure of LarC*_Mt_* in complex with P2TAD, an analog of the intermediate that contains AMP instead of CMP and is capable of being converted to NPMN by LarC*_Lp_* (*17*) (Fig. 3A and fig. S4). Structural superposition of LarC*_Mt_*●P2TAD and LarC*_Mt_* revealed that the overall architecture of the complex was largely unchanged upon P2TAD binding, with an RMSD of 1.3 Å between the two structures (fig. S5A). While no substantial conformational differences were observed, the P2TAD-bound map contained additional density that was absent in the unliganded map (Fig. 3A). Surprisingly, this density appeared in the cleft between the LarC1 and LarC2 domains, where the connecting linker resides, on both sides of the LarC2 hexamer. Inspection of the surrounding protein surface revealed that the density resides within a narrow but elongated interdomain cleft formed by residues from both LarC1 and LarC2 (Fig. 3A and fig. S5B). The cleft extends approximately 30 Å along its longest dimension, closely matching the length of P2TAD (∼25 Å). The density itself was weaker and less well-resolved than the surrounding protein, suggesting low occupancy or conformational heterogeneity. Nevertheless, its size and elongated shape were consistent with P2TAD (Fig. 3B), although the resolution was insufficient to model the molecule precisely.

The putative P2TAD density in the interdomain cleft is positioned near the CTP-binding site in LarC2 but lies on the opposite side of LarC1 from both the histidine cluster and the adjacent pocket lined with conserved acidic residues (Fig. 3, A and B and fig. S5C). Mapping the LarC2 CTP-binding sites, the conserved acidic pockets of LarC1 where nickel insertion is proposed to occur, and the interdomain cleft containing the putative intermediate onto the LarC structure revealed substantial spatial separation between these regions (Fig. 2D and fig. S5C). The center of the putative P2TAD density is located approximately 25 Å from the nearest LarC2 CTP-binding site and approximately 26 Å from the nearest conserved acidic pocket in the proximal LarC1 domain. Thus, the putative P2TAD-binding site is positioned approximately midway between the proposed substrate activation site in LarC2 and the putative nickel insertion site in LarC1.

To probe the functional relevance of the LarC1 pocket and the apparent P2TAD-binding site (Fig. 3C), we generated alanine substitutions for residues in both regions. Substitutions of conserved acidic residues lining the LarC1 pocket near the histidine patch (E104A, D15A, R71A, E119A, D124A, D128A, E187A) markedly reduced activity (Fig. 3D), consistent with prior LarC*_Lp_* studies. In the LarC1-LarC2 cleft, the residues were poorly conserved (fig. S1), with the E243A, E300A, and R323A variants retaining full activity. By contrast, H53A, H226A, and Q325A variants had moderate effects (∼60% compared to wild-type activity), whereas Y180A, N238A, and Q241A substitutions caused more pronounced activity reductions (<20%) (Fig. 3D).

Together, these data reinforce the functional importance of conserved acidic residues in the LarC1 pocket near the putative nickel-binding surface and suggest that the interdomain cleft may serve as a transient intermediate-binding site.

### Structural flexibility of LarC

Our structural and functional analyses indicate that the putative binding sites for P2TCD, both the one near the proposed nickel-binding site and that localized in the LarC1/LarC2 cleft, as well as the CTP-binding site, are spatially separated in the LarC structure. However, these sites must be positioned near each other at some point during catalysis to allow CMPylation of P2TMN and subsequent nickel insertion (Fig. 1A). How these distant sites coordinate is not immediately clear, but it likely involves large conformational changes of the enzyme. Motivated by this hypothesis, we investigated the conformational variability of LarC.

**Fig. 4.**
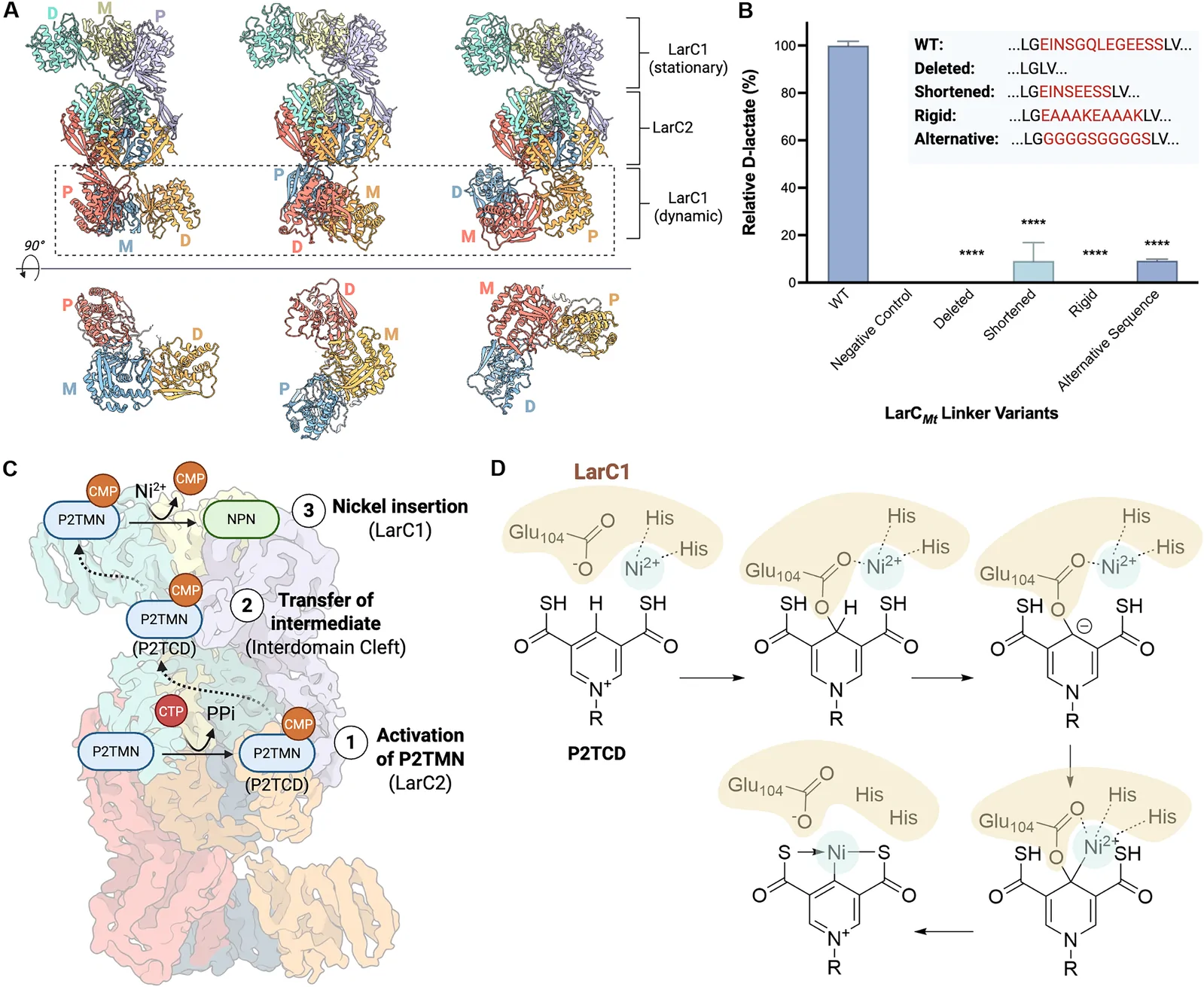
Functional role of the LarC interdomain linker and proposed mechanism. (**A**) Three distinct conformations of full-length LarC observed by cryo-EM, showing rearrangement of the LarC1 trimers relative to the central LarC2 hexamer. The three LarC1 protomers within each trimer are labeled as proximal (P), middle (M), and distal (D) based on their relative proximity to the central LarC2 hexamer. (**B**) Effects of linker deletion, shortening, rigidification, or sequence replacement on LarC activity assessed by lactate racemization assays. Statistical significance was assessed using one-way ANOVA followed by Dunnett’s multiple comparisons test (****P < 0.0001). Each data point represents a technical replicate (*n* = 3 assay replicates per variant). (**C**) Proposed model for LarC catalysis, in which P2TMN activation via CMPylation occurs in the LarC2 domain, followed by transient transfer of the P2TCD intermediate through the interdomain cleft and subsequent nickel insertion in the LarC1 domain. (**D**) Proposed mechanism for nickel insertion by LarC, mediated by transient nucleophilic addition of E104 to the pyridinium ring and neighboring histidine residues in the LarC1 domain.

Multiple rounds of 3D classification during cryo-EM data processing revealed that both LarC*_Mt_* and LarC*_Mt_*●P2TAD adopt the same three conformations (Fig. 4A and fig. S3). Specifically, the LarC1 trimers can assume different orientations relative to the central LarC2 hexamer. Although these conformations give the appearance of ∼120° rotational differences when viewed at the trimer level, closer inspection indicates that they do not arise from rigid-body rotation of a preformed LarC1 trimer. Instead, the conformational variability primarily reflects rearrangements within one LarC1 trimer, while the opposing LarC1 trimer remains largely stationary (Fig. 4A). These rearrangements reposition different LarC1 protomers into the proximal position relative to the LarC2 core (Fig. 4A), indicating that the proximal, middle, and distal designations represent positional states rather than fixed identities of individual subunits. The observed flexibility is likely enabled by the long, flexible linker connecting LarC1 and LarC2 (residues 236–248).

Although these movements do not bring the P2TCD/nickel, LarC1/LarC2 cleft P2TMN/ P2TCD, and CTP binding sites into proximity, the flexibility of this linker suggested it might be functionally important, potentially allowing transient conformational changes not captured in our cryo-EM data. To test the importance of this proposed flexibility, we generated LarC*_Mt_* variants in which the linker was deleted, shortened (from 13 to 7 residues), rigidified (replaced with EAAAKEAAAK), or replaced with a flexible alternative sequence (GGGGSGGGGS). The lactate racemization assay was utilized with each of these LarC*_Mt_* variants after co-expression with LarA*_Lp_*, LarB*_Lp_*, and LarE*_Lp_*, and the results showed that all modifications led to dramatically reduced enzymatic activity (<10% of the wild-type enzyme), supporting a critical role for the linker in LarC function (Fig. 4B). Interestingly, although the linker sequence is not conserved among all LarC homologs (fig. S1), our data suggest that its length, flexibility, and composition all appear important.

Together, these findings raise the possibility that the structural flexibility of LarC, mediated by the interdomain linker, could help facilitate coordination of the spatially separated nickel, substrate, and CTP-binding sites during NPMN cofactor biosynthesis.

## DISCUSSION

Despite its central role in NPMN cofactor biosynthesis, the molecular mechanism of LarC catalysis has remained incompletely understood due to the lack of a full-length structure. Key questions had included the location of the nickel and P2TMN binding sites and how their relative positions to the CTP-binding site could enable CMPylation of P2TMN and subsequent nickel insertion. A previous study resolved high-resolution crystal structures of the LarC2 domain, but the structure of LarC1 domain, and the relative arrangement of LarC1 and LarC2 domains were not known. Our work addressed this gap by determining the first cryo-EM structures of a full-length LarC, using the enzyme from *M. thermoacetica* both in the presence and absence of an intermediate analog, providing a comprehensive view of the intact enzyme and its conformational variability.

Our structures show that LarC assembles as a hexamer, with a central LarC2 hexameric core flanked by two LarC1 trimers. The previously uncharacterized LarC1 domain adopts a unique fold and forms a head-to-tail trimer in which conserved histidine residues cluster to create a histidine-rich surface adjacent to a deep pocket lined with negatively charged, conserved residues that computational modeling studies identify as a potential P2TCD binding site. Functional assays confirm that both the histidine cluster and the acidic pocket are critical for LarC activity. Together, these observations support a model in which the LarC1 pocket serves as the site of nickel insertion.

A second key finding emerged from the LarC*_Mt_*●P2TAD structure. Interestingly, although P2TAD binding did not induce substantial conformational changes in the overall architecture of LarC, our LarC*_Mt_*●P2TAD structure revealed weak additional density in the cleft between LarC1 and LarC2. The size and shape of this density are consistent with P2TAD, suggesting that it represents a binding site for the reaction intermediate. This finding positions the putative nickel insertion site (LarC1 acidic pocket), the putative intermediate-binding site (LarC1/LarC2 interdomain cleft), and the LarC2 CTP-binding site as three spatially separated critical regions of the enzyme, each separated by at least ∼25 Å. Such an arrangement raises an important mechanistic question because CMPylation of P2TMN and subsequent nickel insertion must ultimately be coordinated despite the large distances separating these sites.

One possible clue comes from the conformational flexibility observed in the LarC structure. We observed that the LarC1 trimers can rearrange relative to the LarC2 hexamer, enabled by a long, flexible linker. Our mutagenesis study showed that altering this linker (through deletion, shortening, rigidification, or sequence replacement) dramatically reduces enzymatic activity, suggesting that its length, flexibility, and composition are important for LarC function. Interestingly, while the LarC2 domains form a symmetric hexamer, each LarC1 trimer adopts an asymmetric architecture that generates three non-equivalent LarC1 protomers. As a result, the proximal, middle, and distal LarC1 protomers occupy distinct positions relative to the LarC2 core and differ in their proximity to both the CTP-binding site and the putative intermediate-binding site.

The asymmetric arrangement of the LarC1 trimer becomes particularly intriguing when considered in the context of the three conformations observed by cryo-EM. These conformations effectively reposition different LarC1 protomers into the proximal position relative to the LarC2 core. Because the proximal LarC1 protomer is positioned closest to the interdomain cleft, it is tempting to speculate that this protomer may serve as the primary recipient of the CMPylated intermediate following its formation within LarC2. In this model, the three observed conformations could represent different states within a dynamic conformational landscape in which conformational transitions sequentially reposition neighboring LarC1 protomers into the proximal position. Such conformational cycling could provide a mechanism for coordinating transfer of the intermediate between the CTP-binding site, the interdomain cleft, and the putative nickel insertion site. Although direct evidence for this mechanism is currently lacking, the structural heterogeneity observed in our cryo-EM analysis suggests that conformational rearrangements may contribute to coordinating catalysis across the hexameric assembly.

An additional question raised by the structure concerns the functional significance of the hexameric architecture. Previous studies established that LarC2 forms a hexamer even in the absence of LarC1, but the full-length structure now reveals how this oligomeric scaffold organizes the LarC1 domains around the central catalytic core. One possibility is that hexamerization is required to generate the composite CTP-binding pockets located at LarC2 subunit interfaces.

Based on our structural and functional data, we propose a working model for LarC catalysis (Fig. 4, C and D). In this model, the reaction initiates at the core of the LarC oligomer, within the LarC2 domain, where P2TMN is CMPylated to generate a P2TCD intermediate. The binding site of P2TMN is not known, but the LarC1/LarC2 cleft site may facilitate this interaction. The activated intermediate then transiently engages the interdomain cleft near the LarC1-LarC2 linker, providing a pathway for transfer of the intermediate between the two catalytic regions. Subsequently, P2TCD is repositioned into the conserved acidic pocket within the LarC1 domain, perhaps the proximal one. Within this pocket, E104 is ideally positioned to play a central catalytic role. We propose that E104 acts as a nucleophile that attacks the C4 position of P2TMN, promoting deprotonation at C4 and formation of a carbanion that is stabilized by delocalization over the two adjacent C-C double bonds (Fig. 4D). This step effectively primes the substrate for Ni-C bond formation by poising the carbanion near Ni^2+^ supplied from the adjacent histidine-rich surface. During this step, E104 may also contribute directly to nickel coordination, helping to position the metal productively. Although the exact timing of phosphoanhydride bond hydrolysis in the P2TCD intermediate remains unclear, we postulate that the hydrolysis may drive the pyridinium moiety into close proximity to Ni^2+^, thereby favoring Ni-C bond formation. Following formation of the Ni-C bond, E104 is displaced, with one cofactor thioacid forming an ionic Ni-S bond, while the second thioacid forms a dative bond, yielding the final nickel-bound product. While direct evidence for each step of this pathway remains to be obtained, this model provides a framework that reconciles the spatial separation of binding sites observed in our structures with the known chemistry of NPMN biosynthesis.

This model further raises the possibility that LarC employs large-scale conformational rearrangements to transiently bring spatially separated binding sites into functional proximity during the catalytic cycle. Future studies capturing LarC bound to its natural substrates, P2TMN and nickel, will be essential to test this model and define the sequence of structural transitions that underlie NPMN cofactor biosynthesis. Additionally, the functional and mechanistic relevance of the three distinct LarC1 trimer conformations observed here remains an important open question for future investigation.

## MATERIALS AND METHODS

### Constructs

Bacterial strains, plasmids, and primers used for this study are listed in table S1. PCR amplifications were performed using Vazyme Phanta Flash Super-Fidelity DNA polymerase (Nanjing, China). The primers were purchased from IDT (Newark, NJ, USA) and gene fragments were purchased from Twist Bioscience (South San Francisco, CA, USA). Site-directed mutagenesis and deletion were performed following the in vivo assembly method (*21*). Subcloning of gene fragments was done with the ClonExpress cloning kit (Nanjing, China) following the manufacturer’s manual. The transformations were done according to the standard chemical method using *E. coli* DH5α cells for plasmid amplification (*22*). Plasmid sequencing was submitted to Plasmidsaurus (San Francisco, CA, USA).

Briefly, the gene encoding LarC*_Mt_*, flanked by NdeI and XhoI restriction sites, was synthesized (Integrated DNA Technologies, Coralville, IA, USA). The DNA fragment was inserted into the pLW01 vector (*23*), resulting in expression of LarC*_Mt_* with an N-terminal His_6_-tag followed by a tobacco etch virus (TEV) protease cleavage site.

For the LarC*_Mt_* activity assays, we used the pETDuet and pRSFDuet expression systems (Novagen, Merck KGaA, Darmstadt, Germany) to co-express genes encoding the NPMN cofactor biosynthesis pathway enzymes (LarB*_Lp_*, LarE*_Lp_*, LarC*_Mt_*, and LarA*_Lp_*). The genes encoding LarA*_Lp_* and LarB*_Lp_* were cloned into the pETDuet plasmid. LarA*_Lp_* was engineered to include a C-terminal Strep-tag II. The pRSFDuet plasmid carrying the genes encoding LarA*_Lp_* and LarC*_Mt_* was used as a template for site-directed mutagenesis of *larC* to introduce single alanine substitutions.

### Expression and purification of LarC_*Mt*_

LarC*_Mt_* was overexpressed in *E. coli* BL21 (DE3) (Millipore Sigma) cells grown in TB medium supplemented with 1 mM NiCl_2_ at 37°C until reaching an optical density of 1.0 at 600 nm. The temperature was then lowered to 18°C and protein expression was induced with 1 mM isopropyl β-D-1-thiogalactopyranoside (IPTG) overnight (∼14 hours). Following induction, cells were harvested by centrifugation at 4,000 *g* for 20 min. The resulting bacterial cell pellets were lysed by sonication, and the lysates were ultracentrifuged at 30,000 *g* for 30 min. The clarified lysates were loaded onto a Ni-NTA column, and the resin was washed with a buffer containing 100 mM Tris-HCl (pH 7.5), 300 mM NaCl, 1 mM dithiothreitol (DTT), and 50 mM imidazole to remove nonspecifically bound proteins. LarC*_Mt_* was eluted using a buffer of the same composition adjusted to contain 400 mM imidazole. The elution fractions from the Ni-NTA purification were further purified by size-exclusion chromatography on a Superdex 200 Increase 10/300 GL column (Cytiva) equilibrated in buffer consisting of 100 mM Tris-HCl (pH 8.0), 300 mM NaCl, and 1 mM DTT.

### Expression and purification of LarA_*Lp*_

*E. coli* BL21(DE3) cells were co-transformed with the pRSFDuet plasmid encoding LarE*_Lp_* and LarC*_Mt_* and the pETDuet plasmid encoding LarA*_Lp_* and LarB*_Lp_*. The proteins were overexpressed, and cells were harvested and lysed as described for LarC*_Mt_*. Clarified cell lysates were loaded onto a Strep-Tactin XT 4Flow (IBA) column, and the resin was washed with a buffer containing 100 mM Tris-HCl (pH 7.5), 150 mM NaCl, 0.05 mM Na_2_SO_3_ to remove nonspecifically bound proteins. LarA*_Lp_* was eluted using a buffer of the same composition adjusted to contain 50 mM biotin.

### Lactate racemization assay

The purified Strep II-tagged LarA*_Lp_* was buffer exchanged to remove the Na_2_SO_3_ and biotin from the buffer using a PD-10 desalting column (Marlborough, MA, USA). To assess the LarA*_Lp_* activities, protein samples (1 pmol) were mixed with sodium L-lactate (5–400 mM) in 4-(2-hydroxyethyl)-1-piperazineethanesulfonic acid (HEPES, 100 mM) buffer, pH 7.0, for 1–12 min at 35°C, then heated for 10 min at 95°C to inactivate the enzyme. The precipitated protein was removed by centrifugation at 17,000 x *g* for 10 min and the supernatant was collected. The amount of D-lactate in the sample, produced by the lactate racemase activity of LarA, was measured using a commercial kit (Neogen, Lansing, MI) as previously described (*2*).

### Formation of the LarC*_Mt_*●P2TAD complex

P2TAD (pyridinium-3,5-dithiocarboxylate adenine dinucleotide) was synthesized as previously described (*17*). A tenfold molar excess of P2TAD was added to purified LarC*_Mt_* and incubated for approximately 10 min before cryo-EM grid preparation.

### Cryo-EM sample preparation and data collection

Cryo-EM sample preparation and data collection were performed using procedures similar to those previously described (*24*), with LarC-specific sample conditions and imaging parameters described below. Samples of purified LarC*_Mt_* and the LarC*_Mt_*●P2TAD complex were concentrated to a final concentration of 4.0 mg/ml. An initial cryo-EM trial revealed preferred orientation of the proteins on cryo-EM grids; therefore, n-dodecyl-β-maltoside (DDM) was added to a final concentration of 0.05% (v/v) to mitigate this issue. A 3 μl aliquot of the sample was applied to Quantifoil R1.2/1.3 400-mesh Cu holey carbon grids. The grids were plunge-frozen using a Vitrobot Mark IV (blot time: 3.0 s, wait time: 10 s, blot force: −2). Data collection was performed on a Titan Krios microscope operating at 300 kV at the Life Sciences Institute Cryo-EM Lab at the University of Michigan. For the LarC*_Mt_* dataset, a total of 5,572 movies, each comprising 50 frames, were collected using a Gatan K3 direct electron detector. Images were acquired with a pixel size of 0.85 Å, a total dose of ∼50 e/Å^2^, and a defocus range of −0.8 μm to −2.0 μm. For the LarC*_Mt_*●P2TAD sample, a total of 14,477 movies were collected under the same imaging conditions, including pixel size, total dose, and defocus range.

### Cryo-EM data processing and model building

Cryo-EM data processing and model building followed a previously described workflow, with dataset-specific processing, refinement, and model-building procedures described below (*24*). The initial 5572 movies of the LarC*_Mt_* dataset were aligned and corrected for beam-induced motion using patch motion correction in CryoSPARC (*25*). The contrast transfer function (CTF) parameters were determined using patch CTF estimation in CryoSPARC, and micrographs with CTF fit parameters worse than 10 Å or those showing ice contamination were discarded. A total of 5003 micrographs remained for downstream processing.

Following particle picking and multiple rounds of 2D classification in CryoSPARC to remove poor-quality particles, 535,683 particles were selected for the ab initio model reconstruction algorithm in CryoSPARC and 3D-classification in Relion 4.0 (*26*). The three best 3D classes, consisting of 82,129, 142,648, and 93,068 particles, were reimported to cryoSPARC for a final round of non-uniform refinement, yielding 3.97 Å, 3.85 Å, and 3.87 Å maps based on the gold-standard FSC.

For the LarC*_Mt_*●P2TAD dataset, 14,477 movies were collected and cleaned to 14,260 micrographs after motion correction and CTF estimation using the same procedures described above. Following particle picking and multiple rounds of 2D classification in CryoSPARC to remove poor-quality particles, 2,413,196 particles were selected for the multiclass ab initio model reconstruction*.* The class resembling the LarC architecture (1,542,235 particles) was selected for multiple rounds of 3D classification, resulting in a final subset of 188,303 particles. These particles were refined using a mask encompassing the LarC2 hexamer and a single LarC1 trimer to yield the 3.33 Å map based on the gold-standard FSC.

For atomic model building, the AlphaFold2-predicted LarC*_Mt_* hexamer structure was rigid-body docked into the cryo-EM map using UCSF ChimeraX (*27*). The model was refined using the real-space refinement algorithm in PHENIX and further adjusted manually in COOT (*28*). To optimize the overall model geometry and fit to the experimental cryo-EM map, iterative cycles of real-space refinement in PHENIX and manual adjustments in COOT were performed. The stereochemistry and geometry of the structure were assessed using MolProbity (*29*), and the figures were created using UCSF ChimeraX.

### Structure prediction of the LarC_*Mt*_● P2TCD structure

Structure prediction of the LarC_*Mt*_● P2TCD complex was performed using the Chai-1 online structure-prediction server (*20*). The full-length LarC_*Mt*_ amino acid sequence, together with the SMILES string for P2TCD and the ions Ni^2+^ and Mg^2+^, were used as inputs for modeling. No multiple sequence alignment, structural templates, or specific restraints were applied. Five highly superimposable models were generated, and the top-ranked prediction was used to produce Fig. 2, E and F.

### ConSurf

Evolutionary conservation of LarC*_Mt_* was analyzed with the ConSurf web server (*30*), using the LarC*_Mt_* sequence and the cryo-EM structure as inputs. ConSurf identified homologs, built a multiple sequence alignment, and calculated position-specific conservation scores, which were then mapped onto the structure using the standard ConSurf color scale to highlight conserved residues in the putative active site and ligand-binding site.

## Supplementary Materials

### This PDF file includes:

Figs. S1 to S5 Tables S1 and S2 References

## References

[1] R. P. Hausinger, “Microbial metabolism of nickel” in *Microbial Metabolism of Metals and Metalloids*, C. J. Hurst, Ed. (Springer, 2022), pp. 417–502.

[2] B. Desguin, P. Goffin, E. Viaene, M. Kleerebezem, V. Martin Diaconescu, M. J. Maroney, P. Declercq, P. Soumillion, P. Hols, Lactate racemase is a nickel-dependent enzyme activated by a widespread maturation system. Nat. Commun. 5, 3615 (2014). 24710389 10.1038/ncomms4615 PMC4066177

[3] B. Desguin, T. Zhang, P. Soumillion, P. Hols, J. Hu, R. P. Hausinger, A tethered niacin-derived pincer complex with a nickel-carbon bond in lactate racemase. Science 349, 66–69 (2015). 26138974 10.1126/science.aab2272

[4] B. Desguin, J. Urdiain-Arraiza, M. Da Costa, M. Fellner, J. Hu, T. Desmet, P. Hols, P. Soumillion, Uncovering a superfamily of nickel-dependent hydroxyacid racemases and epimerases. Sci. Rep. 10, 18123 (2020). 33093595 10.1038/s41598-020-74802-6 PMC7583248

[5] S. Chatterjee, S. Gatreddi, S. Gupta, J. L. Nevarez, J. A. Rankin, A. Turmo, J. Hu, R. P. Hausinger, Unveiling the mechanisms and biosynthesis of a novel nickel-pincer enzyme. Biochem. Soc. Trans. 50, 1187–1196 (2022). 35960008 10.1042/BST20220490 PMC9880988

[6] S. Gatreddi, S. Chatterjee, A. Turmo, J. Hu, R. P. Hausinger, A structural view of nickel-pincer nucleotide cofactor-related biochemistry. Crit. Rev. Biochem. Mol. Biol. 59, 402–417 (2024). 39827451 10.1080/10409238.2025.2451443 PMC11925681

[7] B. Desguin, P. Soumillion, P. Hols, R. P. Hausinger, Nickel-pincer cofactor biosynthesis involves LarB-catalyzed pyridinium carboxylation and LarE-dependent sacrificial sulfur insertion. Proc. Natl. Acad. Sci. U.S.A. 113, 5598–5603 (2016). 27114550 10.1073/pnas.1600486113 PMC4878509

[8] A. Turmo, J. Hu, R. P. Hausinger, Characterization of the nickel-inserting cyclometallase LarC from *Moorella thermoacetica* and identification of a cytidinylylated reaction intermediate. Metallomics 14, mfac014 (2022). 35225337 10.1093/mtomcs/mfac014 PMC8962377

[9] B. Desguin, M. Fellner, O. Riant, J. Hu, R. P. Hausinger, P. Hols, P. Soumillion, Biosynthesis of the nickel-pincer nucleotide cofactor of lactate racemase requires a CTP-dependent cyclometallase. J. Biol. Chem. 293, 12303–12317 (2018). 29887527 10.1074/jbc.RA118.003741 PMC6093250

[10] M. Albrecht, Cyclometalation using d-block transition metals: Fundamental aspects and recent trends. Chem. Rev. 110, 576–623 (2010). 20017477 10.1021/cr900279a

[11] R. P. Hausinger, New metal cofactors and recent metallocofactor insights. Curr. Opin. Struct. Biol. 59, 1–8 (2019). 30711735 10.1016/j.sbi.2018.12.008

[12] R. Hausinger, J. Hu, B. Desguin, The nickel-pincer coenzyme of lactate racemase: A case study of uncovering cofactor structure and biosynthesis. Methods Enzymol. 685, 341–371 (2023). 37245907 10.1016/bs.mie.2023.03.006 PMC10626555

[13] N. Caliskan, F. Peske, M. V. Rodnina, Changed in translation: mRNA recoding by −1 programmed ribosomal frameshifting. Trends Biochem. Sci. 40, 265–274 (2015). 25850333 10.1016/j.tibs.2015.03.006 PMC7126180

[14] A. Llamas, R. R. Mendel, G. Schwarz, Synthesis of adenylated molybdopterin: An essential step for molybdenum insertion. J. Biol. Chem. 279, 55241–55246 (2004). 15504727 10.1074/jbc.M409862200

[15] J. Krausze, D. Hercher, M. Zwerschke, W. Kirk, W. Blankenfeldt, R. R. Mendel, T. Kruse, The functional principle of eukaryotic molybdenum insertases. Biochem. J. 475, 1739–1753 (2018). 29717023 10.1042/BCJ20170935 PMC6639804

[16] C. Probst, J. Yang, J. Krausze, W. Hercher, C. P. Richers, T. Spatzal, K. KC, L. J. Gilles, D. C. Rees, R. R. Mendel, M. L. Kirk, T. Kruse, Mechanism of molybdate insertion into pterin-based molybdenum cofactors. Nat. Chem. 13, 758–765 (2021). 34183818 10.1038/s41557-021-00714-1 PMC8325642

[17] T. Vucko, D. Strilets, P. Soumillion, B. Desguin, SP. Vincent, Chemo-enzymatic synthesis of NPN cofactor taking advantage of ADP-ribosyl cyclase and LarC cyclometallase promiscuous activities. Bioorg. Chem. 153, 107879 (2024). 39406107 10.1016/j.bioorg.2024.107879

[18] L. Holm, A. Laiho, P. Törönen, M. Salgado, DALI shines a light on remote homologs: One hundred discoveries. Protein Sci. 32, e4519 (2023). 36419248 10.1002/pro.4519 PMC9793968

[19] M. van Kempen, S. S. Kim, C. Tumescheit, M. Mirdita, J. Lee, C. L. M. Gilchrist, J. Söding, M. Steinegger, Fast and accurate protein structure search with Foldseek. Nat. Biotechnol. 42, 243–246 (2024). 37156916 10.1038/s41587-023-01773-0 PMC10869269

[20] C. Discovery, J. Boitreaud, J. Dent, M. McPartlon, J. Meier, V. Reis, A. Rogozhnikov, K. Wu, Chai-1: Decoding the molecular interactions of life. bioRxiv [Preprint] (2024); 10.1101/2024.10.10.615955.

[21] J. García-Nafría, J. F. Watson, I. H. Greger, IVA cloning: A single-tube universal cloning system exploiting bacterial in vivo assembly. Sci. Rep. 6, 27459 (2016). 27264908 10.1038/srep27459 PMC4893743

[22] “Plasmid transformation of *Escherichia coli* and other bacteria,” in *Methods in Enzymology* (Academic Press, 1991), vol. 204, pp. 63–113; https://www.sciencedirect.com/science/chapter/bookseries/pii/007668799104006A. 10.1016/0076-6879(91)04006-a 1943786

[23] D. Cheng, R. W. Kelley, G. F. Cawley, W. L. Backes, High-level expression of recombinant rabbit cytochrome P450 2E1 in *Escherichia coli* C41 and its purification. Protein Expr. Purif. 33, 66–71 (2004). 14680963 10.1016/j.pep.2003.08.009

[24] D. Granados-Villanueva, A. Rossow, K. H. Kim, Get4/5-mediated remodeling of Get3’s substrate-binding chamber: Insights into tail-anchored protein targeting by the GET pathway. J. Biol. Chem. 301, 110667 (2025). 40902977 10.1016/j.jbc.2025.110667 PMC12506534

[25] A. Punjani, J. L. Rubinstein, D. J. Fleet, M. A. Brubaker, cryoSPARC: Algorithms for rapid unsupervised cryo-EM structure determination. Nat. Methods 14, 290–296 (2017). 28165473 10.1038/nmeth.4169

[26] J. Zivanov, T. Nakane, B. O. Forsberg, D. Kimanius, W. J. Hagen, E. Lindahl, S. H. Scheres, New tools for automated high-resolution cryo-EM structure determination in RELION-3. eLife 7, e42166 (2018). 30412051 10.7554/eLife.42166 PMC6250425

[27] E. C. Meng, T. D. Goddard, E. F. Pettersen, G. S. Couch, Z. J. Pearson, J. H. Morris, T. E. Ferrin, UCSF ChimeraX: Tools for structure building and analysis. Protein Sci. 32, e4792 (2023). 37774136 10.1002/pro.4792 PMC10588335

[28] P. Emsley, K. Cowtan, *Coot*: Model-building tools for molecular graphics. Acta Crystallogr. D Biol. Crystallogr. 60, 2126–2132 (2004). 15572765 10.1107/S0907444904019158

[29] V. B. Chen, W. B. Arendall, J. J. Headd, D. A. Keedy, R. M. Immormino, G. J. Kapral, L. W. Murray, J. S. Richardson, D. C. Richardson, *MolProbity*: All-atom structure validation for macromolecular crystallography. Acta Crystallogr. D Biol. Crystallogr. 66, 12–21 (2010). 20057044 10.1107/S0907444909042073 PMC2803126

[30] H. Ashkenazy, S. Abadi, E. Martz, O. Chay, I. Mayrose, T. Pupko, N. Ben-Tal, ConSurf 2016: An improved methodology to estimate and visualize evolutionary conservation in macromolecules. Nucleic Acids Res. 44, W344–W350 (2016). 27166375 10.1093/nar/gkw408 PMC4987940

[31] A. Turmo, J. Hu, R. P. Hausinger, Characterization of the nickel-inserting cyclometallase LarC from *Moorella thermoacetica* and identification of a CMPylated reaction intermediate. Metallomics 14, mfac014 (2022). 35225337 10.1093/mtomcs/mfac014 PMC8962377

[32] J. L. Nevarez, A. Turmo, S. Gatreddi, S. Gupta, J. Hu, R. P. Hausinger, Overcoming barriers for investigating nickel-pincer nucleotide cofactor-related enzymes. MBio 16, e03404–e03424 (2024). 39679682 10.1128/mbio.03404-24 PMC11796402

