# Supplementary material for: Structural and functional analysis of LarC, a CTP-dependent cyclometallase required for nickel-pincer nucleotide cofactor biosynthesis
Source: Sci Adv. 2026 Aug 26;12(35):eaeh6867. doi: 10.1126/sciadv.aeh6867 (PMC13510736; doi:10.1126/sciadv.aeh6867)
Supplement: Supplementary file 1 [file sciadv.aeh6867_sm.pdf]

Science Advances
AAAS

Supplementary Materials for

Structural and functional analysis of LarC, a CTP-dependent cyclometallase required for nickel-pincer nucleotide cofactor biosynthesis

Robert Wolfe *et al.*

Corresponding author: Kelly H. Kim, kimhyoj6@msu.edu; Robert P. Hausinger, hausinge@msu.edu; Jian Hu, hujian1@msu.edu

Sci. Adv. **12**, eaeh6867 (2026)
DOI: 10.1126/sciadv.aeh6867

This PDF file includes:

Figs. S1 to S5
Tables S1 and S2
References

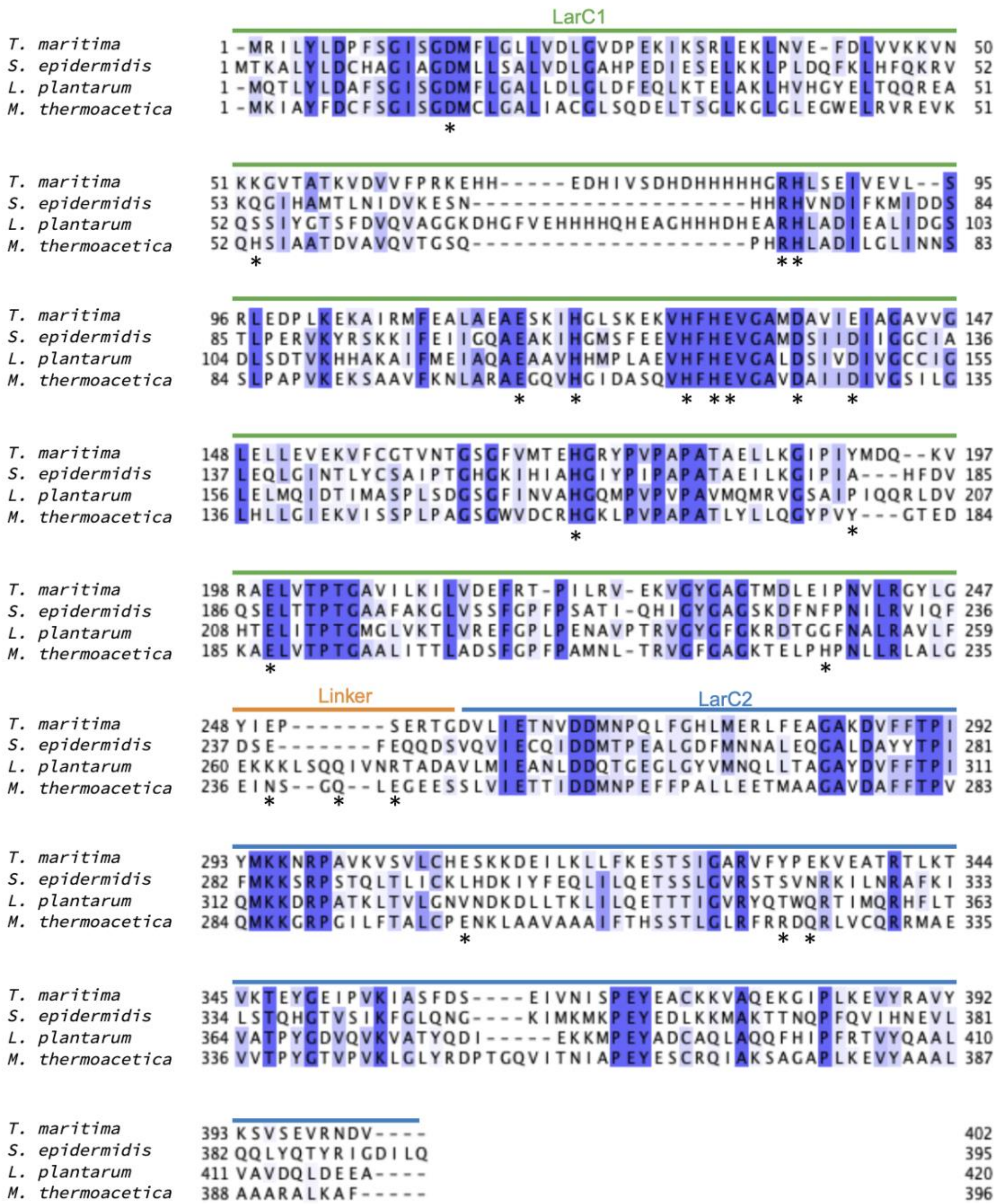

Fig. S1. Multiple sequence alignment of LarC. Multiple sequence alignment of LarC proteins from various bacterial species is shown, with conserved residues highlighted. Positions marked with an asterisk (*) correspond to the residues that were subjected to single alanine substitutions in this study.

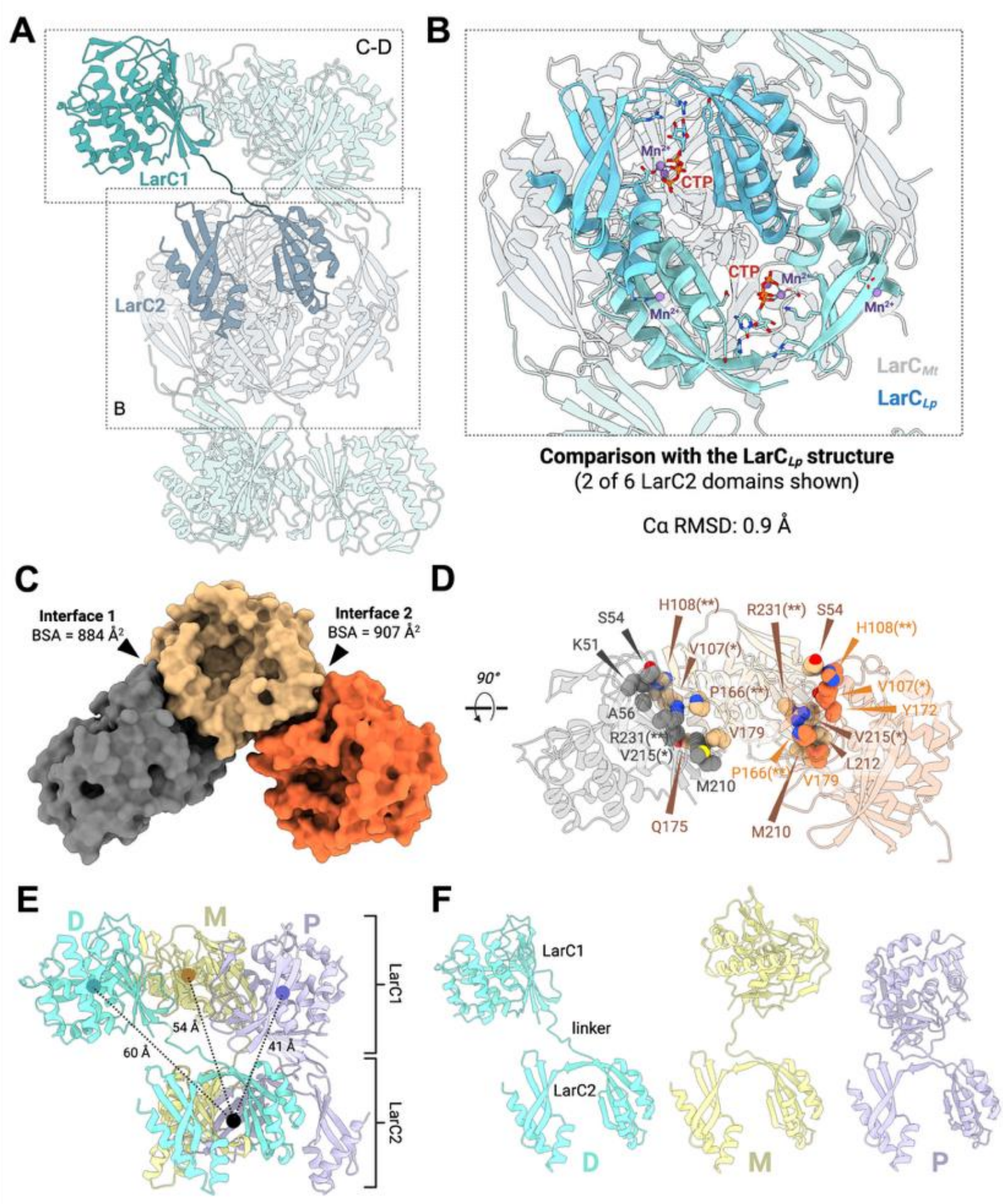

Fig. S2. LarC oligomeric assembly and interaction interfaces. (A) Overall hexameric assembly of LarC. One protomer is shown in solid color, while the remaining subunits are displayed in faded colors for clarity. **(B)** Structural comparison of the LarC2 hexamer from LarC*Mt* overlaid with the previously solved CTP-bound LarC*Lp* crystal structure (PDB: 6BWQ). For clarity, only two of the six subunits of LarC*Lp* are shown (blue and light blue). The bound CTP molecules and Mn^{2+} ion are shown, along with the coordinating residues depicted in stick representation. **(C)** LarC1 trimer with each monomer colored distinctly. The two interaction interfaces are indicated by arrows, along with the corresponding buried surface area (BSA). **(D)** Interaction interface within the LarC1 trimer. Residues contributing to the interface are shown in sphere representation. Residues marked with * denote moderately conserved residues, whereas ** indicates strongly conserved residues. **(E)** LarC1 protomers are designated as distal (D), middle (M), and proximal (P) according to their relative proximity to the LarC2 hexamer. Distances between the centers of the LarC1 protomers and the LarC2 core are indicated. **(F)** Isolated LarC1-LarC2 protomers from the distal, middle, and proximal positions illustrating the distinct orientations adopted by the LarC1 domains relative to the LarC2 core and the connecting linker.

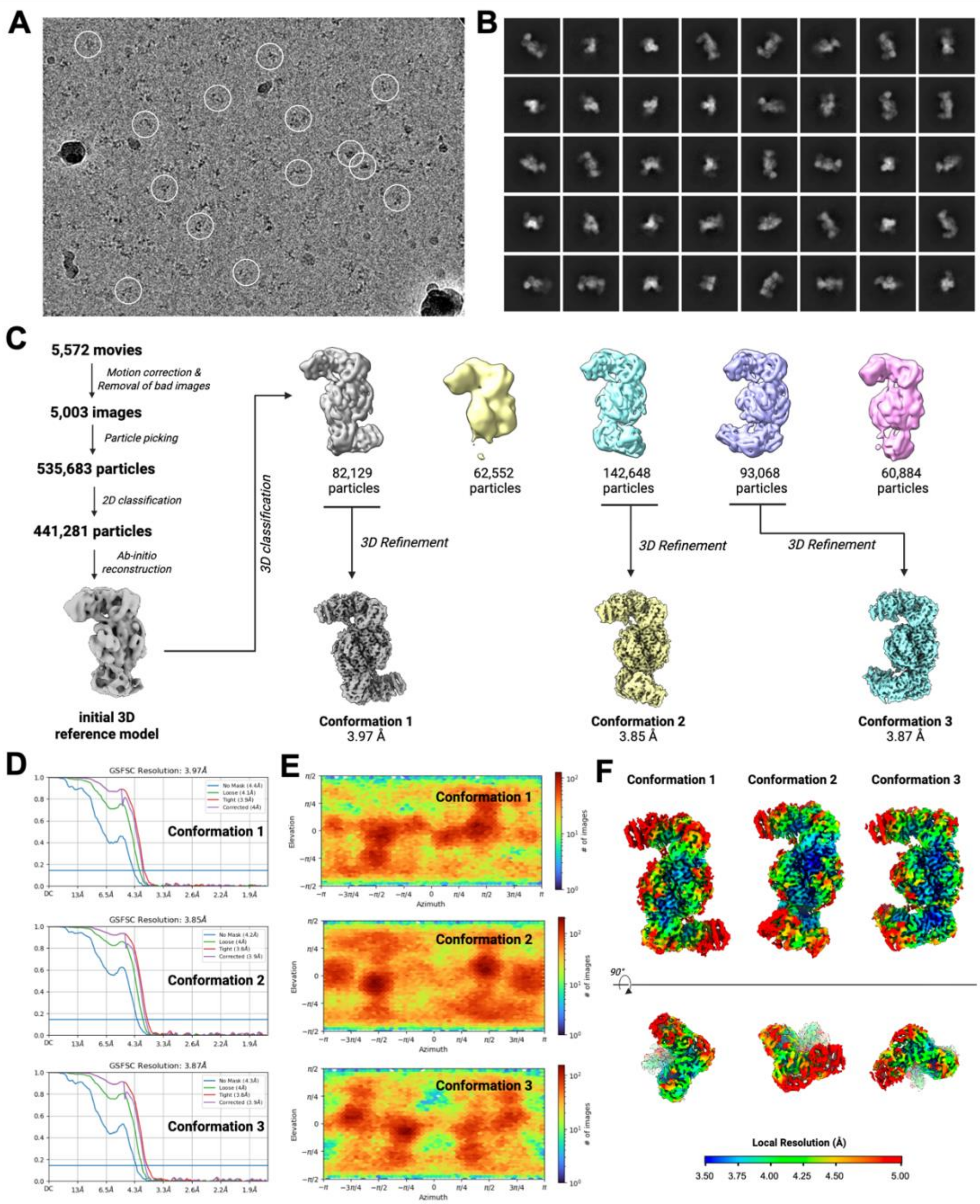

Fig. S3. Cryo-EM data processing workflow for LarC$_{Mt}$. (A) Representative cryo-EM micrograph showing particles of LarC$_{Mt}$ embedded in vitreous ice. **(B)** Selected 2D class averages, revealing distinct orientations and structural features. **(C)** Data processing workflow used to determine the high-resolution cryo-EM structures of LarC$_{Mt}$. **(D)** Fourier shell correlation (FSC) curves used to estimate the resolution of the final 3D reconstruction. FSC curves are shown for unmasked, spherical mask, loose mask, and tight mask conditions. The 0.143 cutoff criterion is indicated by the horizontal bue line. **(E)** Particle orientation distribution plot for the final cryo-EM reconstruction. **(F)** Final 3D cryo-EM reconstructions of LarC$_{Mt}$ colored by local resolution as estimated by CryoSPARC. The color scale bar indicates resolution in angstroms

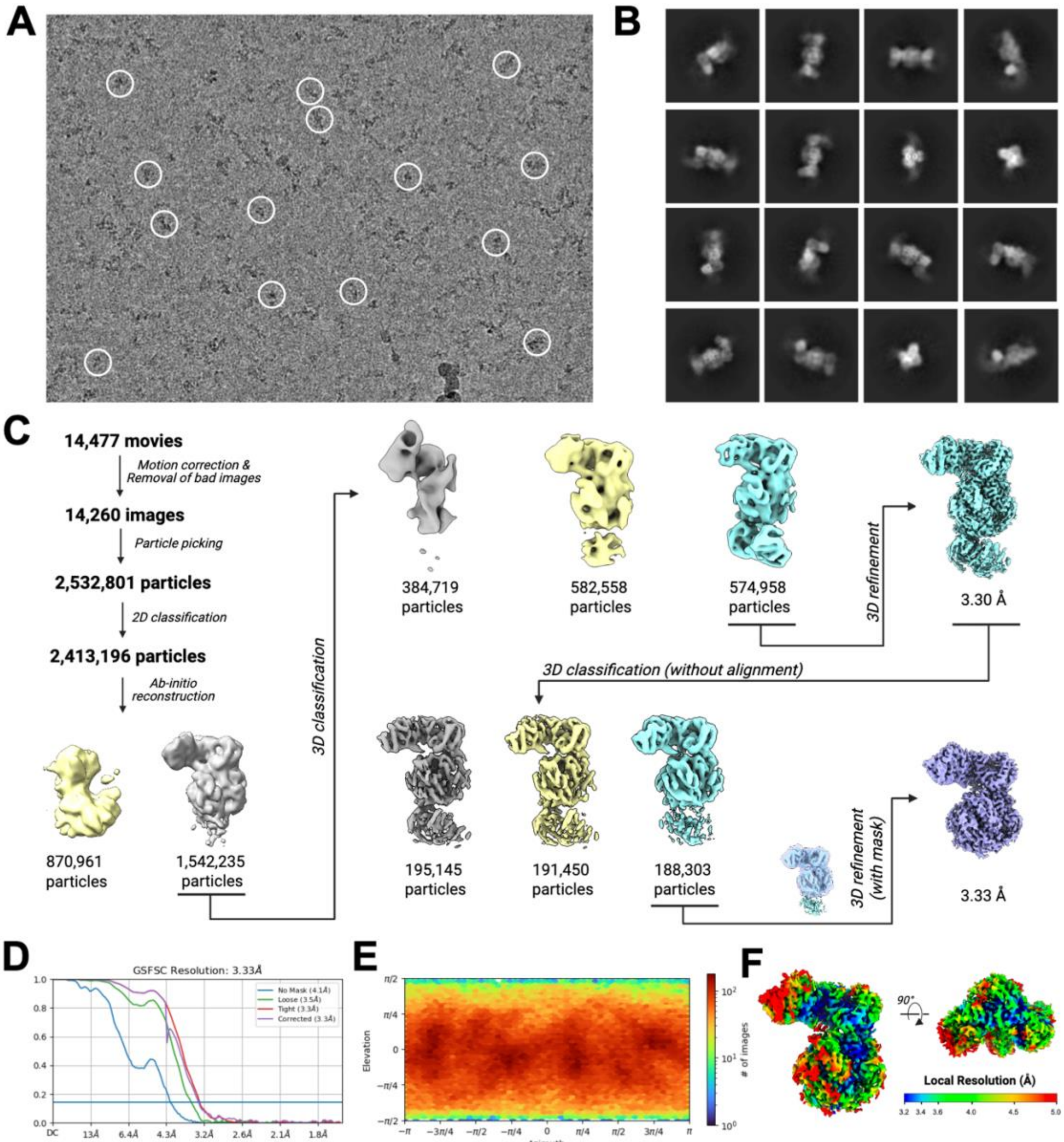

Fig. S4. Cryo-EM data processing workflow for LarC$_{Mt}$●P2TAD. (A) Representative cryo-EM micrograph showing particles of LarC$_{Mt}$●P2TAD embedded in vitreous ice. **(B)** Selected 2D class averages, revealing distinct orientations and structural features. **(C)** Data processing workflow used to determine the high-resolution cryo-EM structure of LarC$_{Mt}$●P2TAD. **(D)** Fourier shell correlation (FSC) curves used to estimate the resolution of the final 3D reconstruction. FSC curves are shown for unmasked, spherical mask, loose mask, and tight mask conditions. The 0.143 cutoff criterion is indicated by the horizontal blue line. **(E)** Particle orientation distribution plot for the final cryo-EM reconstruction. **(F)** Final 3D cryo-EM reconstruction of LarC$_{Mt}$●P2TAD colored by local resolution as estimated by CryoSPARC. The color scale bar indicates resolution in angstroms.

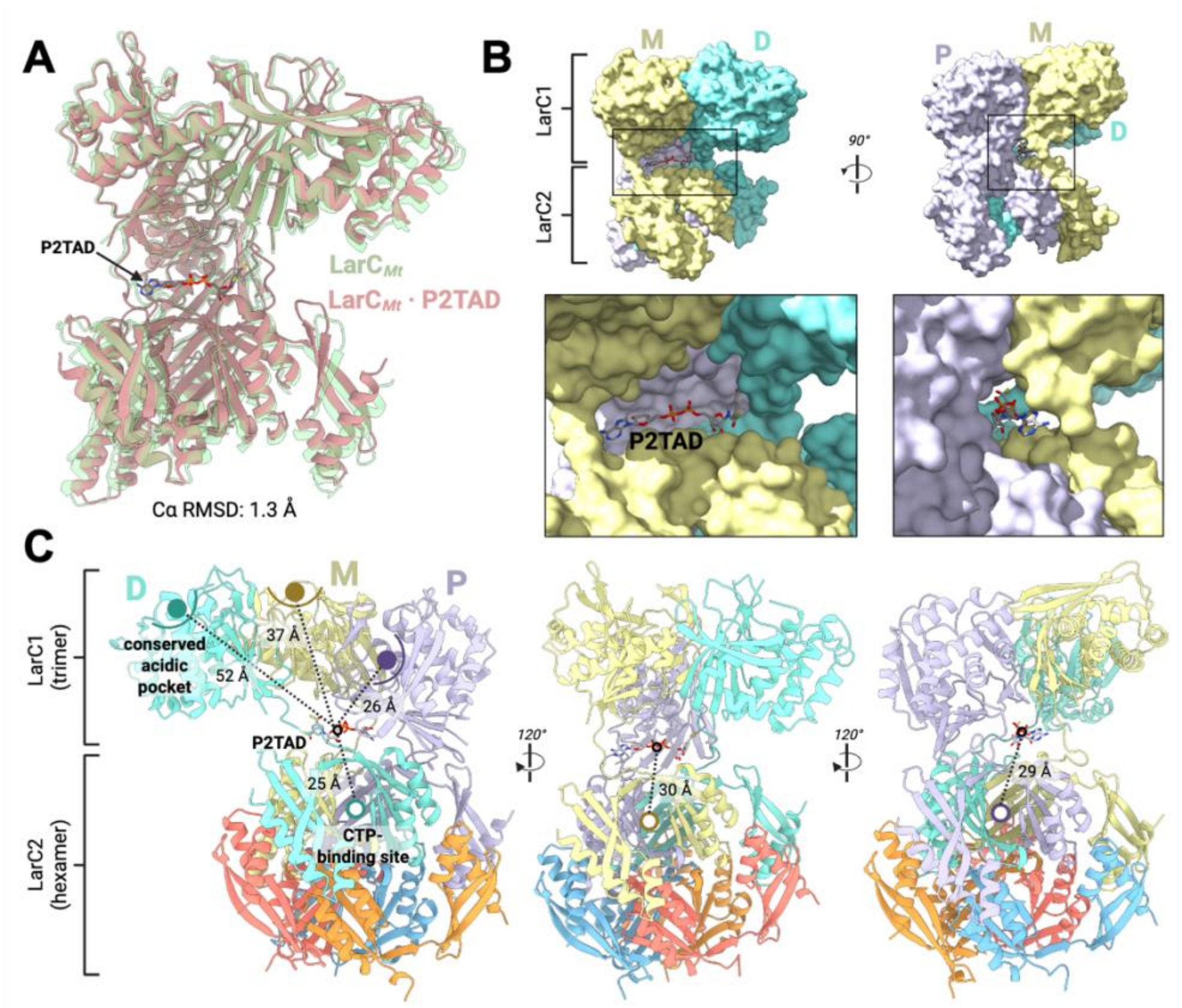

Fig. S5. Identification of a putative intermediate-binding site in the LarC1/LarC2 interdomain cleft. (A) Structural superposition of substrate-free LarC$_{Mt}$ (green) and LarC$_{Mt}$●P2TAD (pink). **(B)** Surface representation of the LarC$_{Mt}$●P2TAD structure showing the location of P2TAD within the interdomain cleft formed between LarC1 and LarC2. LarC1 protomers are labeled as proximal (P), middle (M), and distal (D). Insets show enlarged views of the P2TAD binding site. **(C)** Spatial relationship between the LarC2 CTP-binding site, the putative P2TAD-binding site in the LarC1/LarC2 interdomain cleft, and the conserved acidic pockets of the proximal, middle, and distal LarC1 protomers. Distances between the putative P2TAD-binding site, the LarC2 CTP-binding site, and the conserved acidic pockets are indicated.

Table S1. List of strains, plasmids, and primers

Strain, plasmid or primer	Characteristic(s) or sequence	Source or reference
**<u>Strains</u>**		
E. coli		
DH5α	F– ϕ80lacZΔ M15 Δ (*lacZYA-argF*) *U169 recA1 endA1 hsdR17* (rK– mK+) *phoA supE44* λ- *thi–1 gyrA96 relA1*	ThermoFisher
**<u>Plasmids</u>**		
pLWO1-HR-LarC$_{Mt}$	*Amp*^{r}; His-TEV-LarC$_{Mt}$	Turmo, 2022 (31)
pETDuet-LarAB$_{Lp}$	*Amp*^{r}; LarA$_{Lp}$-Strep and LarB$_{Lp}$	Nevarez, 2024 (32)
pRSFDuet-LarE$_{Lp}$LarC$_{Mt}$	*Kan*^{r}; LarE$_{Lp}$ and LarC$_{Mt}$	Nevarez, 2024 (32)
pRSFDuet-LarE$_{Lp}$LarC$_{Mt}$-Ala-variants	*Kan*^{r}; LarE$_{Lp}$ and LarC$_{Mt}$ alanine variant (20 total)	This study
pRSFDuet-LarE$_{Lp}$LarC$_{Mt}$-linker-variants	*Kan*^{r}; LarE$_{Lp}$ and LarC$_{Mt}$ linker variants (4 total)	This study
**<u>Primers</u>**	The orientation is 5’ to 3’ in all cases.	
fw_D15A	GCG GTA TTA GCG GTG CGA TGT GTC TGG GTG	Site-directed mutagenesis
rv_D15A	ACC GCT AAT ACC GCT AAA GCA ATC AAA ATA GGC G	Site-directed mutagenesis
fw_H53A	CGTGAAGTTAAACAGgcgAGCATTGCAGCAACCG	Site-directed mutagenesis
rv_H53A	CTG TTT AAC TTC ACG AAC ACG CAG TTC CCA ACC TTC	Site-directed mutagenesis
fw_R71A	CCGGTAGCCAGCCGCATgcgCATCTGGCAGATATTC	Site-directed mutagenesis
rv_R71A	ATGCGGCTGGCTACCGGTAACCTGAACTGCAACATCGGTTG	Site-directed mutagenesis
fw_H72A	GCCAGCCGCATCGTgcgCTGGCAGATATTC	Site-directed mutagenesis
rv_H72A	ACGATGCGGCTGGCTACCGGTAAC	Site-directed mutagenesis
fw_H108A	GCGGAAGGTCAGGTTgcgGGTATTGATGCAAG	Site-directed mutagenesis
rv_H108A	AACCTGACCTTCCGCACGTGCCAGG	Site-directed mutagenesis
fw_H116A	GATGCAAGCCAGGTTgcgTTTCATGAAGTTGGTG	Site-directed mutagenesis
rv_H116A	AACCTGGCTTGCATCAATACCATGAACCTG	Site-directed mutagenesis

fw_H118A	CAAGCCAGGTTCATTTTgcgGAAGTTGGTGCAG	Site-directed mutagenesis
rv_H118A	AAA ATG AAC CTG GCT TGC ATC AAT ACC ATG AAC CTG AC	Site-directed mutagenesis
fw_E119A	GCCAGGTTCATTTTCATgcgGTTGGTGCAGTTG	Site-directed mutagenesis
rv_E119A	ATG AAA ATG AAC CTG GCT TGC ATC AAT ACC ATG AAC CTG	Site-directed mutagenesis
fw_D124A	GAAGTTGGTGCAGTTgcgGCCATTATTGATATTG	Site-directed mutagenesis
rv_D124A	AAC TGC ACC AAC TTC ATG AAA ATG AAC CTG GC	Site-directed mutagenesis
fw_D128A	GTTGATGCCATTATTgcgATTGTTGGTAGC	Site-directed mutagenesis
rv_D128A	AAT AAT GGC ATC AAC TGC ACC AAC TTC ATG	Site-directed mutagenesis
fw_H160A	GTT GAT TGT CGT GCG GGT AAA CTG CCG GTT C	Site-directed mutagenesis
rv_H160A	ACG ACA ATC AAC CCA ACC GCT ACC GGC AGG C	Site-directed mutagenesis
fw_Y180A	GCAGGGTTATCCGGTTgcgGGCACCGAAG	Site-directed mutagenesis
rv_Y180A	AAC CGG ATA ACC CTG CAG CAG ATA CAG TGT TGC CGG AG	Site-directed mutagenesis
fw_E187A	CACCGAAGATAAAGCAgcgCTGGTTACCCCG	Site-directed mutagenesis
rv_E187A	TGCTTTATCTTCGGTGCCATAAACCGGATAACCCTG	Site-directed mutagenesis
fw_H226A	CAGGTAAAACCGAACTGCCGgcgCCGAATCTGC	Site-directed mutagenesis
rv_H226A	CGG CAG TTC GGT TTT ACC TGC ACC AAA ACC AAC ACG GGT C	Site-directed mutagenesis
fw _N238A	CTG GCA CTG GGT GAA ATC GCG AGC GGT CAG C	Site-directed mutagenesis
rv_N238A	GAT TTC ACC CAG TGC CAG ACG CAG CAG ATT CGG ATG	Site-directed mutagenesis
fw_E243A	CAA TAG CGG TCA GCT GGC GGG TGA AGA AAG CAG	Site-directed mutagenesis
rv_E243A	CAG CTG ACC GCT ATT GAT TTC ACC CAG TGC CAG AC	Site-directed mutagenesis
fw_delete-linker	GCTGCGTCTGGCACTGGGTCTGGTTATTGAAACCACCATTG	Deletion
rv_delete-inker	ACCCAGTGCCAGACGCAGCAGATTCGGATGCGGCAG	Deletion
fw_truncate-linker	CACTGGGTGAAATCAATGAAGAAAGCAGCCTGGTTATTG	Deletion

rv_truncate-linker	ATTGATTTCACCCAGTGCCAGACGCAGCAGATTCGGATGC	Deletion
fw_linkswap_vector	GTTTACCGCACTGTGTCCGGAAAATAAACTGGCAGCA	Insertion
rv_linkswap_vector	GTGTGGTAATCAGTGCGGCACCGGTCGGGG	Insertion
fw_linkswap	CCCCGACCGGTGCCGCACTGATTACCACACTGG	Insertion
rv_linkswap	AGTTTATTTTCCGGACACAGTGCGGTAAACA	Insertion
**<u>Gene Fragments</u>**	The orientation is 5’ to 3’ in all cases.	
(GGGGS)x2 linker	CACTGATTACCACACTGGCCGATAGCTTTGGTCCGTTTCCGGCAATG<br>AATCTGACCCGTGTTGGTTTTGGTGCAGGTAAAACCGAACTGCCGCA<br>TCCGAATCTGCTGCGTCTGGCACTGGGTGGAGGAGGAAGTGGTGGT<br>GGTGGAAGCAGCCTGGTTATTGAAACCACCATTGATGATATGAACCC<br>GGAATTTTTTCCGGCACTGCTGGAAGAAACCATGGCAGCCGGTGCC<br>GTGGATGCATTTTTTACACCGGTTCAGATGAAAAAAGGTCGTCCGG<br>GTATTCTGTTTACCGCACTGTG	
(EAAAK)x2 linker	GCCGCACTGATTACCACACTGGCCGATAGCTTTGGTCCGTTTCCGGC<br>AATGAATCTGACCCGTGTTGGTTTTGGTGCAGGTAAAACCGAACTGC<br>CGCATCCGAATCTGCTGCGTCTGGCACTGGGTGAAGCGGCGGCAAA<br>AGAAGCAGCCGCTAAGCTGGTTATTGAAACCACCATTGATGATATGA<br>ACCCGGAATTTTTTCCGGCACTGCTGGAAGAAACCATGGCAGCCGG<br>TGCCGTGGATGCATTTTTTACACCGGTTCAGATGAAAAAAGGTCGTC<br>CGGGTATTCTGTTTACCGCAC	

Table S2. Cryo-EM data collection and refinement statistics

	**LarC$_{Mt}$ (Conformation 1)** EMD-76279 PDB: 12BI	**LarC$_{Mt}$ (Conformation 2)** EMD-76280 PDB: 12BJ	**LarC$_{Mt}$ (Conformation 3)** EMD-76281 PDB: 12BK	**P2TAD●LarC$_{Mt}$** EMD-76282 PDB: 12BL
Data collection and processing				
Voltage (kV)	300	300	300	300
Electron exposure (e/Å^2)	50	50	50	50
Defocus (μm)	-0.8 to -2.0	-0.8 to -2.0	-0.8 to -2.0	-0.8 to -2.0
Pixel size (Å)	0.8487	0.8487	0.8487	0.834
Symmetry	C1	C1	C1	C1
Initial particles	441,281	441,281	441,281	2,413,196
Final particles	82,129	142,648	93,068	188,303
Map resolution (Å)	3.97	3.85	3.87	3.33
FSC threshold	0.143	0.143	0.143	0.143
Refinement				
Model composition				
Protein residues	2390	2386	2376	1653
Non-hydrogen atoms	17852	17818	17730	12399
Ligands	0	0	0	1
Average B-factor (Å^2)	84.11	105.57	157.36	50.44
R.m.s. deviations (RMSD)				
Bond lengths (Å)	0.006	0.004	0.003	0.004
Bond angles (Å)	0.864	0.684	0.585	0.732
Validation				
MolProbity score	1.77	1.43	1.31	1.50
Overall mean Q-Score	0.75	0.74	0.74	0.73
Clash score	8.84	7.27	5.76	9.32
Poor rotamers (%)	0.48	0.16	0.11	0.31
Ramachandran plot				
Favored (%)	95.79	97.85	98.48	97.99
Allowed (%)	4.21	2.15	1.52	2.01
Outliers (%)	0	0	0	0

REFERENCES

1. R. P. Hausinger, "Microbial metabolism of nickel" in *Microbial Metabolism of Metals and Metalloids*, C. J. Hurst, Ed. (Springer, 2022), pp. 417–502.

2. B. Desguin, P. Goffin, E. Viaene, M. Kleerebezem, V. Martin Diaconescu, M. J. Maroney, P. Declercq, P. Soumillion, P. Hols, Lactate racemase is a nickel-dependent enzyme activated by a widespread maturation system. *Nat. Commun.* **5**, 3615 (2014).

3. B. Desguin, T. Zhang, P. Soumillion, P. Hols, J. Hu, R. P. Hausinger, A tethered niacin-derived pincer complex with a nickel-carbon bond in lactate racemase. *Science* **349**, 66–69 (2015).

4. B. Desguin, J. Urdiain-Arraiza, M. Da Costa, M. Fellner, J. Hu, T. Desmet, P. Hols, P. Soumillion, Uncovering a superfamily of nickel-dependent hydroxyacid racemases and epimerases. *Sci. Rep.* **10**, 18123 (2020).

5. S. Chatterjee, S. Gatreddi, S. Gupta, J. L. Nevarez, J. A. Rankin, A. Turmo, J. Hu, R. P. Hausinger, Unveiling the mechanisms and biosynthesis of a novel nickel-pincer enzyme. *Biochem. Soc. Trans.* **50**, 1187–1196 (2022).

6. S. Gatreddi, S. Chatterjee, A. Turmo, J. Hu, R. P. Hausinger, A structural view of nickel-pincer nucleotide cofactor-related biochemistry. *Crit. Rev. Biochem. Mol. Biol.* **59**, 402–417 (2024).

7. B. Desguin, P. Soumillion, P. Hols, R. P. Hausinger, Nickel-pincer cofactor biosynthesis involves LarB-catalyzed pyridinium carboxylation and LarE-dependent sacrificial sulfur insertion. *Proc. Natl. Acad. Sci. U.S.A.* **113**, 5598–5603 (2016).

8. A. Turmo, J. Hu, R. P. Hausinger, Characterization of the nickel-inserting cyclometallase LarC from *Moorella thermoacetica* and identification of a cytidinylylated reaction intermediate. *Metallomics* **14**, mfac014 (2022).

9. B. Desguin, M. Fellner, O. Riant, J. Hu, R. P. Hausinger, P. Hols, P. Soumillion, Biosynthesis of the nickel-pincer nucleotide cofactor of lactate racemase requires a CTP-dependent cyclometallase. *J. Biol. Chem.* **293**, 12303–12317 (2018).

10. M. Albrecht, Cyclometalation using d-block transition metals: Fundamental aspects and recent trends. *Chem. Rev.* **110**, 576–623 (2010).

11. R. P. Hausinger, New metal cofactors and recent metallocofactor insights. *Curr. Opin. Struct. Biol.* **59**, 1–8 (2019).

12. R. Hausinger, J. Hu, B. Desguin, The nickel-pincer coenzyme of lactate racemase: A case study of uncovering cofactor structure and biosynthesis. *Methods Enzymol.*, **685**, 341–371 (2023).

13. N. Caliskan, F. Peske, M. V. Rodnina, Changed in translation: mRNA recoding by −1 programmed ribosomal frameshifting. *Trends Biochem. Sci.* **40**, 265–274 (2015).

14. A. Llamas, R. R. Mendel, G. Schwarz, Synthesis of adenylated molybdopterin: An essential step for molybdenum insertion. *J. Biol. Chem.* **279**, 55241–55246 (2004).

15. J. Krausze, D. Hercher, M. Zwerschke, W. Kirk, W. Blankenfeldt, R. R. Mendel, T. Kruse, The functional principle of eukaryotic molybdenum insertases. *Biochem. J.* **475**, 1739–1753 (2018).

16. C. Probst, J. Yang, J. Krausze, W. Hercher, C. P. Richers, T. Spatzal, K. KC, L. J. Gilles, D. C. Rees, R. R. Mendel, M. L. Kirk, T. Kruse, Mechanism of molybdate insertion into pterin-based molybdenum cofactors. *Nat. Chem.* **13**, 758–765 (2021).

17. T. Vucko, D. Strilets, P. Soumillion, B. Desguin, SP. Vincent, Chemo-enzymatic synthesis of NPN cofactor taking advantage of ADP-ribosyl cyclase and LarC cyclometallase promiscuous activities. *Bioorg. Chem.* **153**, 107879 (2024).

18. L. Holm, A. Laiho, P. Törönen, M. Salgado, DALI shines a light on remote homologs: One hundred discoveries. *Protein Sci.* **32**, e4519 (2023).

19. M. van Kempen, S. S. Kim, C. Tumescheit, M. Mirdita, J. Lee, C. L. M. Gilchrist, J. Söding, M. Steinegger, Fast and accurate protein structure search with Foldseek. *Nat. Biotechnol.* **42**, 243–246 (2024).

20. C. Discovery, J. Boitreaud, J. Dent, M. McPartlon, J. Meier, V. Reis, A. Rogozhnikov, K. Wu, Chai-1: Decoding the molecular interactions of life. bioRxiv [Preprint] (2024); https://doi.org/10.1101/2024.10.10.615955.

21. J. García-Nafría, J. F. Watson, I. H. Greger, IVA cloning: A single-tube universal cloning system exploiting bacterial *in vivo* assembly. *Sci. Rep.* **6**, 27459 (2016).

22. "Plasmid transformation of *Escherichia coli* and other bacteria," in *Methods in Enzymology* (Academic Press, 1991), vol. 204, pp. 63–113; https://www.sciencedirect.com/science/chapter/bookseries/pii/007668799104006A.

23. D. Cheng, R. W. Kelley, G. F. Cawley, W. L. Backes, High-level expression of recombinant rabbit cytochrome P450 2E1 in *Escherichia coli* C41 and its purification. *Protein Expr. Purif.* **33**, 66–71 (2004).

24. D. Granados-Villanueva, A. Rossow, K. H. Kim, Get4/5-mediated remodeling of Get3's substrate-binding chamber: Insights into tail-anchored protein targeting by the GET pathway. *J. Biol. Chem.* **301**, 110667 (2025).

25. A. Punjani, J. L. Rubinstein, D. J. Fleet, M. A. Brubaker, cryoSPARC: Algorithms for rapid unsupervised cryo-EM structure determination. *Nat. Methods* **14**, 290–296 (2017).

26. J. Zivanov, T. Nakane, B. O. Forsberg, D. Kimanius, W. J. Hagen, E. Lindahl, S. H. Scheres, New tools for automated high-resolution cryo-EM structure determination in RELION-3. *eLife* **7**, e42166 (2018).

27. E. C. Meng, T. D. Goddard, E. F. Pettersen, G. S. Couch, Z. J. Pearson, J. H. Morris, T. E. Ferrin, UCSF ChimeraX: Tools for structure building and analysis. *Protein Sci.* **32**, e4792 (2023).

28. P. Emsley, K. Cowtan, *Coot*: Model-building tools for molecular graphics. *Acta Crystallogr. D Biol. Crystallogr.* **60**, 2126–2132 (2004).

29. V. B. Chen, W. B. Arendall, J. J. Headd, D. A. Keedy, R. M. Immormino, G. J. Kapral, L. W. Murray, J. S. Richardson, D. C. Richardson, *MolProbity*: All-atom structure validation for macromolecular crystallography. *Acta Crystallogr. D Biol. Crystallogr.* **66**, 12–21 (2010).

30. H. Ashkenazy, S. Abadi, E. Martz, O. Chay, I. Mayrose, T. Pupko, N. Ben-Tal, ConSurf 2016: An improved methodology to estimate and visualize evolutionary conservation in macromolecules. *Nucleic Acids Res.* **44**, W344–W350 (2016).

31. A. Turmo, J. Hu, R. P. Hausinger, Characterization of the nickel-inserting cyclometallase LarC from *Moorella thermoacetica* and identification of a CMPylated reaction intermediate. *Metallomics* **14**, mfac014 (2022).

32. J. L. Nevarez, A. Turmo, S. Gatreddi, S. Gupta, J. Hu, R. P. Hausinger, Overcoming barriers for investigating nickel-pincer nucleotide cofactor-related enzymes. *MBio* **16**, e03404–24 (2024).
